# Thermoelectric transport in molecular crystals driven by gradients of thermal electronic disorder

**DOI:** 10.1126/sciadv.adr1758

**Published:** 2024-10-23

**Authors:** Jan Elsner, Yucheng Xu, Elliot D. Goldberg, Filip Ivanovic, Aaron Dines, Samuele Giannini, Henning Sirringhaus, Jochen Blumberger

**Affiliations:** ^1^ Department of Physics and Astronomy and Thomas Young Centre, University College London, London WC1E 6BT, UK.; ^2^ Cavendish Laboratory, University of Cambridge, Cambridge, CB3 0HE, UK.; ^3^ Institute for the Chemistry of OrganoMetallic Compounds, National Research Council (ICCOM-CNR), I-56124 Pisa, Italy.

## Abstract

Thermoelectric materials convert a temperature gradient into a voltage. This phenomenon is relatively well understood for inorganic materials but much less so for organic semiconductors (OSs). These materials present a challenge because the strong thermal fluctuations of electronic coupling between the molecules result in partially delocalized charge carriers that cannot be treated with traditional theories for thermoelectricity. Here, we develop a quantum dynamical simulation approach revealing in atomistic detail how the charge carrier wave function moves along a temperature gradient in an organic molecular crystal. We find that the wave function propagates from hot to cold in agreement with the experiment, and we obtain a Seebeck coefficient in good agreement with experimental measurements that are also reported in this work. Detailed analysis reveals that gradients in thermal electronic disorder play an important role in determining the magnitude of the Seebeck coefficient, opening unexplored avenues for the design of OSs with improved Seebeck coefficients.

## Teaser

The mechanism of thermoelectric transport in organic crystals is revealed by nonadiabatic molecular dynamics simulation.

## INTRODUCTION

Organic semiconductors (OSs) have emerged as promising materials for thermoelectric applications (*1*–*3*). Recent studies have shown that relatively high *ZT* figure of merit values are achievable (*ZT* = *T*α^2^σ/κ), where *T* is the temperature, α is the Seebeck coefficient, σ is the electronic conductivity, and κ is the thermal conductivity, for example, *ZT* = 0.42 has been measured in the doped conducting polymer PEDOT:PSS [poly(3,4-ethylenedioxythiophene):polystyrene sulfonate] at room temperature (*4*). The thermoelectric properties of a single-molecule break junction have also recently been characterized, highlighting the potential of molecules in thermoelectric applications due to their chemical tunability (*5*). The combination of good thermoelectric properties with intrinsic mechanical flexibility in molecular materials opens up a range of possibilities, for example, in wearable devices, a rapidly growing industry where the need for batteries or external charging could be eliminated (*6*). As such, there is a need for a detailed fundamental understanding of thermoelectric transport in OSs that can aid the interpretation of experiments and inform the design of improved organic thermoelectric materials.

Here, we aim to establish a molecular-scale understanding of thermoelectricity and the Seebeck coefficient, α, in high-mobility OSs. The Seebeck coefficient quantifies the open-circuit voltage, Δ*V*_oc_, developed in response to an applied temperature difference, Δ*T*, α = −Δ*V*_oc_/Δ*T*. For wide-band inorganic semiconductors, α is usually computed in the framework of coherent transport theories, e.g., Landauer theory or the Boltzmann transport equation (*7*–*9*). The latter has also been used for OSs (*10*, *11*) and superconductors (*12*). For narrow-band semiconductors, where charge transport occurs via incoherent hopping of localized charge carriers, Heikes formula (*13*, *14*) or Emin’s theory (*15*–*17*) may be used instead. It is now well established, however, that the transport scenario for ordered OSs is in between these two limiting extremes (*18*–*37*). Charge carriers partially delocalize over the molecular units of these materials due to sizable electronic couplings, while their delocalization is limited by intermolecular electron-phonon couplings. Transient localization theory has been derived specifically for this intermediate regime and has been successful in predicting charge carrier mobilities as well as in providing design rules for this class of materials (*19*, *20*). Yet, a theoretical description of thermoelectricity in this regime is still relatively unexplored.

Computer simulations, specifically atomistic mixed quantum-classical molecular dynamics (MD) such as fragment orbital–based surface hopping (FOB-SH) (*25*, *34*–*37*) and similar implementations (*26*–*28*) have given important complementary molecular-level insight into the charge transport process in ordered OSs, largely supporting the assertions of transient localization theory. In FOB-SH, the quantum dynamics of an excess charge carrier in the valence or conduction band of the semiconductor is propagated under the influence of time-dependent classical nuclei according to Tully’s fewest switches surface hopping (*38*). This method is particularly well suited for the simulation of charge transport in the difficult intermediate transport regime where relevant transport parameters, notably electronic coupling and reorganization energy, are on the same order of magnitude, as is the case for high-mobility OSs. Applications of FOB-SH to ordered OSs have shown that thermal electronic excitations give rise to expansion and contraction events of the charge carrier wave function, also denoted “transient delocalizations,” which result in charge displacement, diffusion, and mobility. However, it remains unknown how the important effect of transient delocalizations plays out in a system subject to a temperature gradient. What is the microscopic origin that drives charge carriers across a temperature gradient in an OS? How can the directional charge flow and the Seebeck coefficient be enhanced?

To investigate these questions, we abandon the open-circuit condition, i.e., zero charge flow, for which Seebeck coefficients are usually measured or computed, and simulate the real-time propagation of the charge carrier wave function using FOB-SH in an OS subjected to a temperature gradient, representing short-circuit conditions in the experiment. We choose single-crystalline rubrene for this purpose, an experimentally well characterized high-mobility OS where hole transport is thought to occur in the transient delocalization (TD) regime (*18*, *35*–*37*). We observe a net migration of the hole wave function from hot to cold indicative of the Seebeck effect and in agreement with the experiment. We show that the directional motion of the hole wave function is due to gradients in thermal electronic disorder. This causes the carrier wave function to transition with higher probability to a neighboring electronic state on the cold side compared to the hot side, resulting in the movement from hot to cold.

Our results are analyzed in terms of the general expression for current density in the presence of gradients in temperature, chemical potential, and electrical potential (see Eq. 1 below). In this framework, the Seebeck coefficient, α, can be written as the sum of three contributions: a kinetic contribution due to the temperature gradient-induced current or drift velocity, α_v_; a thermodynamic contribution due to the temperature gradient-induced change in chemical potential, α_c_; and an electric field contribution, α_e_ (α = α_v_ + α_c_ + α_e_). Under short-circuit conditions, all terms contribute in general, whereas under open-circuit conditions, only the thermodynamic and electric field terms contribute and the kinetic term is zero. α_c_ and α_e_ are independent of transport mechanism and have been the sole focus of most computational studies (*16*, *39*–*41*), while the kinetic term depends on the charge transport mechanism and, to our best knowledge, has eluded a rigorous calculation so far owing to the complex nature of the TD mechanism described above.

Simulations without an external electric field (α_e_ = 0), representing short-circuit conditions in the experiment, show that the kinetic contribution to the Seebeck coefficient is substantial $\left(\alpha_{v}>\frac{k_{B}}{e}\right)$, on the same order of magnitude as, albeit somewhat smaller than, the thermodynamic contribution α_c_. These results are consolidated by simulations with an electric field that is chosen to cancel the kinetic contribution, representing the open-circuit condition in the experiment, reproducing the Seebeck coefficient obtained without an external electric field. On the basis of these results, we discuss viable strategies for increasing the Seebeck coefficient of thermoelectric materials in the regime of TD.

## RESULTS

### Molecular model

**Fig. 1.**
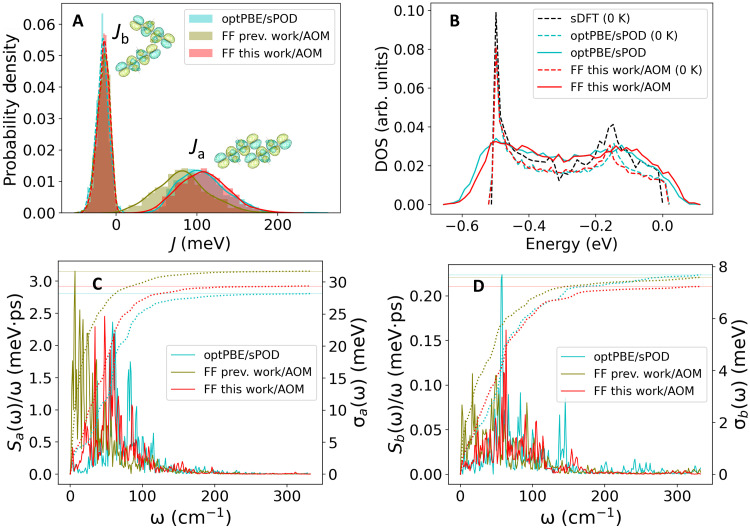
Validation of optimized force field (FF this work) and fast electronic coupling calculation (AOM) used in FOB-SH simulations of rubrene against ab initio calculations. (**A**) Thermal distributions of electronic couplings along crystallographic directions *a* and *b* of rubrene, *J_a_* and *J_b_*, at 300 K. optPBE/sPOD denotes ab initio MD simulation of rubrene crystal using the optPBE functional (*47*) with electronic couplings calculated along the trajectory according to the sPOD (*48*) method using PBE. FF prev. work/AOM and FF this work/AOM denote classical MD simulation of the rubrene crystal with dihedral parameters used in (*35*, *36*) and reoptimized for this work (see Supplementary Note S1), respectively, with electronic couplings calculated along the trajectory using the AOM (*45*, *46*). (**B**) Normalized density of states (DOS) from the Kohn-Sham Hamiltonian at the PBE level (sDFT, black) and from the valence band Hamiltonian (Eq. 4) with electronic couplings obtained from optPBE/sPOD (blue) or FF this work/AOM (red). Site energies were sampled from a Gaussian distribution centered on zero with variance $\sigma_{E}^{2}=k_{B}T\lambda$, λ = 0.152 eV is the DFT reorganization energy for hole transfer (*35*). Dashed lines correspond to optimized structures (0 K), and solid lines correspond to sampled configurations at 300 K. In each case, the peak of the DOS was aligned with the peak of the sDFT DOS at −0.499 eV relative to the top of the valence band. arb. units, arbitrary units. (**C** and **D**) Spectral density functions from the cosine transform of the autocorrelation function of the *J_a_* and *J_b_* time series (solid) and accumulated frequency resolved root mean square fluctuations of *J_a_* and *J_b_*, σ*_a_* and σ*_b_*, respectively (*63*).

In the FOB-SH method (*42*–*44*) that will be used for simulation of thermoelectric transport, the electronic Hamiltonian for hole transport is constructed in a site basis of highest occupied molecular orbitals (HOMOs) with site energies obtained from force fields and electronic couplings between the site-localized orbitals from an ultrafast coupling estimator denoted analytic overlap method (AOM) (*45*, *46*) (see Materials and Methods for details). The latter are also sometimes denoted diabatic electronic couplings or transfer integrals, and we simply refer to them here as electronic couplings. Using such an approximate but computationally efficient scheme, it is vital to demonstrate that properties governing charge transport are well captured when compared to higher-level electronic structure methods. We have used a force field with optimized dihedral parameters for rubrene, alongside optimized AOM electronic coupling parameters (denoted FF this work/AOM in Fig. 1). We validate their performance by comparison with results from ab initio MD simulation [using the optPBE van der Waals density functional (*47*)] and scaled projector operator–based diabatization (sPOD) method (*48*) for electronic couplings (denoted optPBE/sPOD in Fig. 1). We find that the electronic coupling distributions (Fig. 1A) and the density of valence band states at *T* = 0 and 300 K (Fig. 1B) are very well reproduced. Moreover, the spectral density functions of the electronic couplings related to off-diagonal electron-phonon coupling compare well with the ab initio MD results both in terms of frequency (0 to 200 cm^−1^) and intensity (panels C and D). The force field we have previously used for rubrene (*35*, *36*) (denoted FF prev. work/AOM) underestimates couplings along the high-mobility direction (*a*) and gives red-shifted spectral density functions. Both deficiencies are cured with the improved dihedral parameters. We report numerical values for couplings and coupling fluctuations obtained using the different approaches in table S2. A description of the updated force field parameters and a more detailed discussion of results shown in Fig. 1 are presented in Supplementary Notes S1 and S2, respectively.

### Temperature dependence of hole transport

**Fig. 2.**
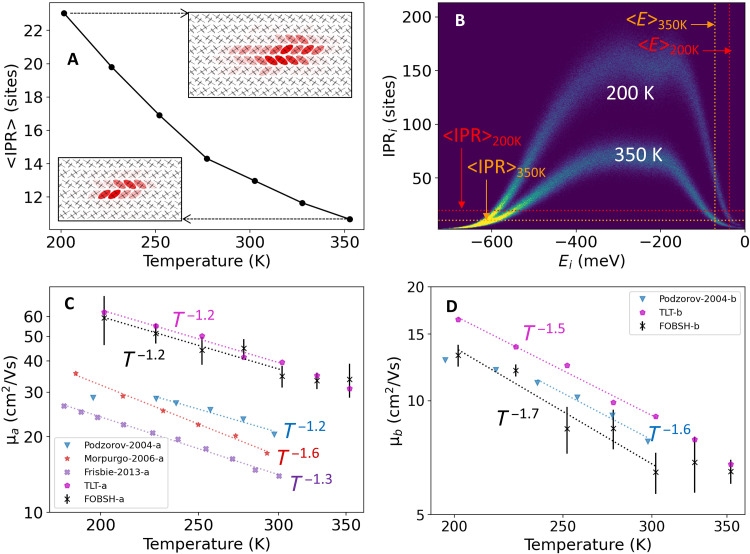
Temperature dependences of IPR, density of valence band states, and hole mobility in rubrene. (**A**) Time-averaged IPR of the hole wave function Ψ(*t*) (Eq. 11), 〈IPR〉, showing increasing localization with increasing temperature. (**B**) Density of valence band states (increasing from dark blue to yellow) with respect to their IPR (Eq. 12) and energy ($E_{k}^{\text{ad}}$, eigenvalues of Eq. 4) relative to the energy at the valence band maximum, for *T* = 200 K and *T* = 350 K. Data were averaged over configurations from MD simulations run at the specified temperature. Mean values for the IPR of the hole wave function (Eq. 11) and for the potential energy of the hole (∑*_k_*∣*u_k_*∣^2^*E_k_*) from FOB-SH simulations are shown in dashed lines. (**C** and **D**) Hole mobility along the *a* and *b* directions, μ*_a_* and μ*_b_*, respectively, from FOB-SH simulation and Eq. 10 (black) and experimental data from (*50*) (blue), (*66*) (red), and (*67*) (purple). Hole mobilities from transient localization theory (TLT, eq. S6 in Supplementary Note S8) are in magenta. Best fits μ ∝ *T*^−*n*^ are indicated in dashed lines. Error bars were calculated by partitioning the total number of trajectories into five blocks (≈130 trajectories per block) and taking the SD of the block-averaged mobilities.

To set the scene for hole transport subject to a temperature gradient, we first present results for simulations at constant temperatures in the range of 200 to 350 K. The lattice parameters were adjusted for each temperature to account for the small thermal expansion of the crystal as observed in the experiment (*49*). The FOB-SH simulations of hole transport follow a protocol very similar to the one established in previous works (*35*, *36*) except that the improved force field is used (see Materials and Methods for details). The results obtained are presented in Fig. 2, and numerical values are summarized in Table 1.

**Table 1.** Summary of temperature-dependent properties of rubrene obtained from FOB-SH simulations at constant temperature unless noted otherwise.

*T* (K)	〈*J_a_*〉^*^	σ_a_^*^	〈*J_b_*〉^*^	σ_b_^*^	σ_E_^*^	〈IPR〉^†^	σ_IPR_^†^	τ*_r_*^‡^	*t_r_* ^‡^
200	113.4	23.7	−17.6	5.7	51.2	23.0	16.2	88.4	11.9
225	112.0	24.9	−17.2	6.0	54.5	19.8	14.1	83.6	11.0
250	110.6	26.1	−16.7	6.2	57.2	16.9	12.6	81.2	10.4
275	108.5	27.2	−16.1	6.5	60.4	14.3	10.7	76.4	9.8
276^§^	107.6	27.3	−15.9	6.4	59.3	13.7	10.0		
300	106.8	28.2	−15.6	6.7	63.5	13.0	9.6	72.8	9.1
300^§^	106.8	28.2	−15.6	6.6	62.8	12.5	9.4		
325	105.2	29.2	−15.2	6.8	65.1	11.6	8.8	71.5	9.0
325^§^	106.1	29.0	−15.4	6.8	64.7	11.7	8.8		
350	103.5	30.1	−14.7	7.0	68.3	10.7	8.1	69.1	8.6

*Mean value of electronic couplings, 〈*J*_*a*(*b*)_〉, and root mean square fluctuations of electronic couplings, σ_*a*(*b*)_, and site energies, σ*_E_*, in meV.

†Average IPR, 〈IPR〉 (Eq. 11) and corresponding root mean square fluctuations, σ_IPR_, of the hole wave function.

‡Average time between two TD events, τ*_r_* (fs), defined as configurations where the IPR of a trajectory is greater than 〈IPR〉 + σ_IPR_ and average duration of a TD event, *t_r_* (fs).

**Fig. 3.**
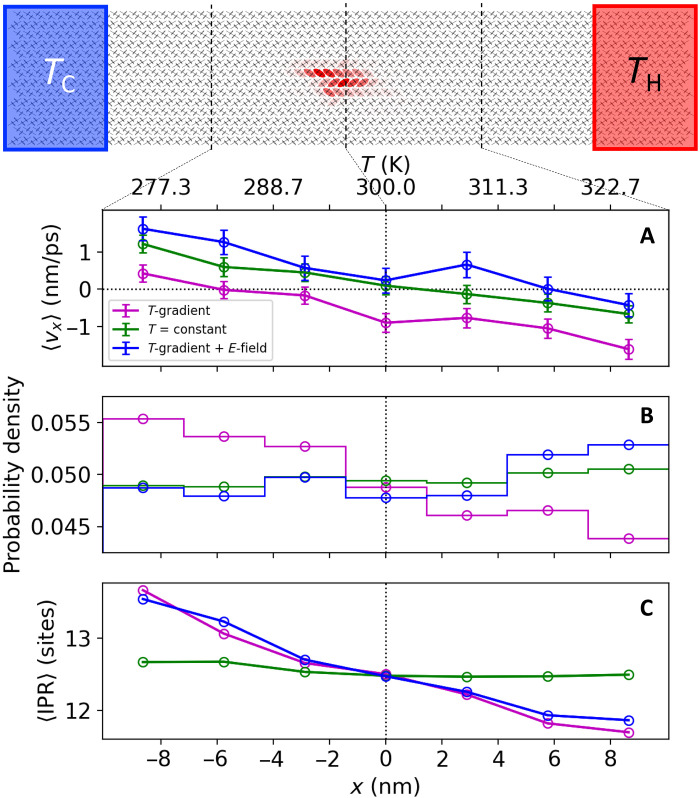
Thermoelectric hole transport in rubrene from FOB-SH. The graphic at the top shows the simulation box with heat baths on the cold and hot sides indicated. A snapshot of the hole wave function Ψ as it moves from the hot to the cold end is depicted by superposing red ellipses on each molecular site with opaqueness proportional to the site population ∣*u_k_*∣^2^. Position-resolved quantities obtained from FOB-SH simulations under a temperature gradient (∂*_x_T* = 2.8 K/nm, magenta), under a temperature gradient and an external electric field (∂*_x_T* = 2.8 K/nm and −∂*_x_*ϕ = 3 × 10^3^ V/cm, blue), and at constant temperature (*T* = 300 K, green) are shown in (A) to (C) for the central 20 nm of the simulation cell centered around 300 K (region indicated in black dashed lines). The data were binned using bin widths of 2.9 nm and averaged over all time steps and FOB-SH trajectories. (**A**) Drift velocity of the COC (Eq. 13) of the hole wave function Ψ, 〈*v_x_*〉 (see Materials and Methods for details of calculation). Error bars correspond to the SE of the velocity distribution, $\sigma_{v}\left(x\right)/\sqrt{N\left(x\right)}$, where σ_v_(*x*) is the SD of velocities observed in bin *x* and *N*(*x*) is the number of data points. (**B**) Probability density of the COC of the hole wave function. (**C**) Average IPR of the hole wave function (Eq. 11), 〈IPR〉.

§From FOB-SH simulation under a temperature gradient ∂*_x_T* = 2.8 K/nm for bins of length of 2.9 nm centered at the indicated temperature (same binning procedure as in Fig. 3).

Figure 2A shows that the hole wave function propagated by FOB-SH becomes increasingly localized as the temperature increases. The average inverse participation ratio (IPR) of the wave function decreases from 23 molecules at 200 K to 13 and 11 molecules at 300 and 350 K, respectively. The same trend can be seen in the IPR-resolved density of electronic valence band states that make up the hole wave function, shown for 200 and 350 K in Fig. 2B. The average delocalization of valence band states at a given energy is notably smaller at 350 K than at 200 K due to the increased dynamic disorder of the electronic Hamiltonian. The width of the thermal distribution of electronic couplings, i.e., off-diagonal electron-phonon coupling, σ*_a_* and σ*_b_*, are about 25% higher at 350 K than at 200 K, while the width in the distribution of site energies, i.e., diagonal electron-phonon coupling, σ*_E_*, increases by around 33% over the same range (see Table 1). The mean values of electronic couplings slightly decrease with temperature due to the small thermal expansion of the crystal (see Table 1 and Supplementary Note S3). The maximum of the IPR is shifted toward the top of the valence band at both temperatures, a consequence of the overall positive electronic coupling sign combination in the rubrene crystal, sgn(*P*,*T*_1_,*T*_2_) = sgn(*J_a_*, *J_b_*, *J_b_*) = (+, −, −) (*20*, *37*), but the height of the maximum is markedly reduced at the higher temperature. Although the hole occupies more highly excited (= lower energy) valence band states at the higher temperature, the average IPR is substantially lower than at the lower temperature (compare horizontal dashed lines in Fig. 2B). The same holds for the hole wave function propagated by FOB-SH (panel A) as it closely samples a Boltzmann distribution of the valence band states in the long-time limit (*25*).

The hole mobility obtained from the time-dependent mean square displacement (MSD) of the hole wave function (see Materials and Methods) is shown in Fig. 2 (C and D) as a function of temperature, with fits to μ ∼ *T*^−*n*^. Good agreement of the theoretical and experimental data to the power law decay fits indicates a band-like temperature dependence of mobility. We obtain exponents *n* =1.2 for mobility along *a* and *n* =1.7 for mobility along *b*, which is within the relatively narrow experimental range of estimates. Our absolute mobilities are in good agreement with results from transient localization theory (see Supplementary Note S8 for details) but about a factor of 1.5 to 2 higher than the experimental values. For instance, our predicted room temperature mobility along the high-mobility direction *a* is 34.8 ± 3.5 cm^2^ V^−1^ s^−1^ compared to ~20 cm^2^ V^−1^ s^−1^ reported by Podzorov *et al.* (*50*). This difference may be due to factors not accounted for in the FOB-SH simulations, for example, remaining structural disorder or chemical impurities in the crystalline samples or surface and finite carrier concentration effects.

The mechanism of charge transport is TD at all temperatures [see our previous work for a detailed description (*36*)]. We define a TD event as a period in time where the IPR is larger than a threshold given by 〈IPR〉 + σ_IPR_, where 〈IPR〉 and σ_IPR_ are the mean and the width of the thermal distribution of the IPR, respectively (*35*). We find that both the average duration of a TD event, *t_r_*, and the average time between two subsequent TD events, τ*_r_*, decrease with increasing temperature (Table 1), but the effect is rather small. Such a behavior is expected because TD events are associated with transitions (surface hops) between electronic valence band states and the frequency of such transitions increases with increasing kinetic energy. The small reduction in τ*_r_* would lead to an increase in mobility with increasing temperature, but this effect is smaller than the decrease in mobility due to increasing hole localization.

### Thermoelectric transport

To study the thermoelectric transport using FOB-SH, we require a computational protocol for simulating a uniform temperature gradient. This was achieved by defining local heat baths at specified temperatures, maintained through thermostatting (*51*). Thermal bath regions were defined at *T* = 250 K and *T* = 350 K, separated by 36 nm along the *a* direction (50 unit cells), resulting in a stable and linear temperature profile (see figs. S8 and S9). We note that such a temperature gradient is about three orders of magnitude larger than realizable in the experiment although necessary in our simulations to ensure convergence of statistical sampling of drift velocity (see below). The hole wave function in FOB-SH simulations was initialized on a single molecule at five different initial positions, uniformly spaced along the *x* direction of the active region spanning the “hot” and the “cold” end of the simulation cell (400 trajectories each). All properties obtained from FOB-SH, including drift velocity, were averaged over all sets of starting positions. We also performed a control simulation using the same setup as above except that the temperature of both thermal baths was set equal to 300 K, i.e., constant room temperature simulation. Further details regarding the computational setup are provided in Materials and Methods and Supplementary Note S10.

Figure 3 shows the position-resolved average drift velocity of the center of charge (COC) of the hole wave function (panel A), probability density of the COC (panel B), and average IPR (panel C) for positions within ±10 nm with respect to the middle of the simulation cell (at position *x* = 0, *T* = 300 K) spanning temperatures between 275 and 325 K. In the control simulations at constant temperature (data in green), the probability density of COC and the average IPR are approximately the same at all positions. The average drift velocity at *x* = 0 is zero, as expected. The average drift velocity should vanish at all positions for a constant temperature profile, but in practice, this is not the case. We observe a small linear change in position-dependent drift velocity as one approaches the hard boundaries at the hot and the cold side of the simulation cell. At the boundaries, the charge carrier gets reflected and this results in a boundary force, leading to a small drift velocity pointing toward the middle of the simulation cell. In the center of the simulation cell, the artificial boundary effects cancel and the average drift velocity vanishes.

In the presence of the temperature gradient (data in magenta), the drift velocities shift fairly uniformly to more negative values compared to the constant temperature simulations. At the center of the cell where no artificial boundary effects are present, we obtain 〈*v_x_*〉 = −0.89 ± 0.25 nm ps^−1^, corresponding to a net motion of the charge carrier from the hot to the cold region, i.e., the thermoelectric effect. We emphasize that the thermoelectric effect obtained from FOB-SH is not due to skewed initial conditions—the same number of trajectories are initialized from hot and cold regions and from the middle (see above). Rather, the hole wave function moves, on average, faster from hot to cold than from cold to hot, giving rise to a net current and an increase in probability density of the hole on the cold side (panel B).

**Fig. 4.**
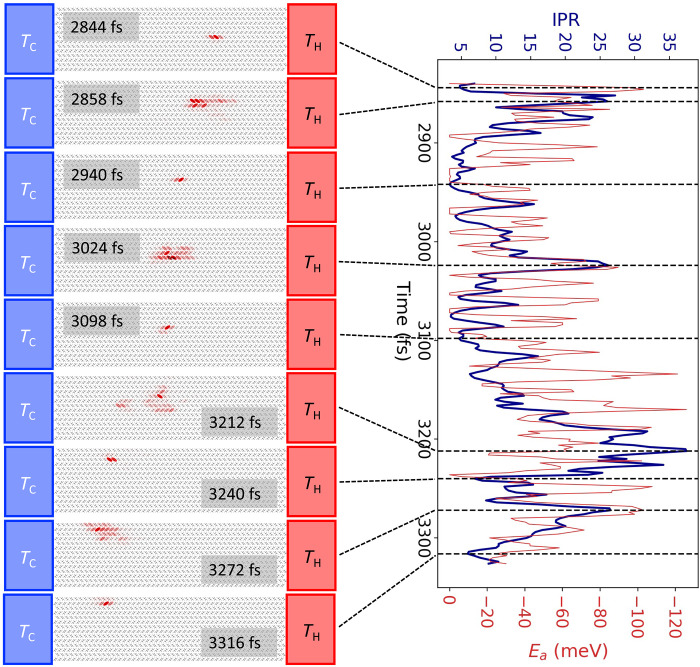
Snapshots of the charge carrier wave function, Ψ, along a single FOB-SH trajectory as it moves from hot to cold. Ψ (Eq. 3) is represented by superposing red ellipses onto each molecular site with opaqueness proportional to the site population, ∣*u_k_*∣^2^. The IPR of the carrier wave function (Eq. 11) and the energy of the active adiabatic state, *E_a_*, are plotted on the RHS in dark blue and red, respectively.

A representative FOB-SH trajectory of a hole injected in the hot region and moving toward the cold region is shown in Fig. 4. We find that the transport occurs via a series of TD events, similarly to the case of constant temperature. However, on average, the hole wave function steadily expands as it moves from hot to cold [see the position-resolved average IPR in Fig. 3C (data in magenta)]. The average IPR at a given temperature along the temperature gradient simulations is very similar to the corresponding value obtained in a constant temperature simulation at this temperature. This is due to mean electronic couplings, root mean square fluctuations of electronic couplings (i.e., off-diagonal electronic disorder), and root mean square fluctuations of site energies (i.e., diagonal electronic disorder) along the temperature gradient being very similar too (see Table 1). Hence, the trends discussed above for temperature-dependent delocalization and electronic disorder carry over, in a quasicontinuous manner, to the system under a temperature gradient. The spatial heterogeneity of wave function delocalization under a temperature gradient is a feature of the TD regime in contrast to the standard assumption that spatial variation caused by the temperature gradient is related only to the spreading out of the Fermi-Dirac distribution, i.e., transport level of carriers (*8*, *52*).

**Fig. 5.**
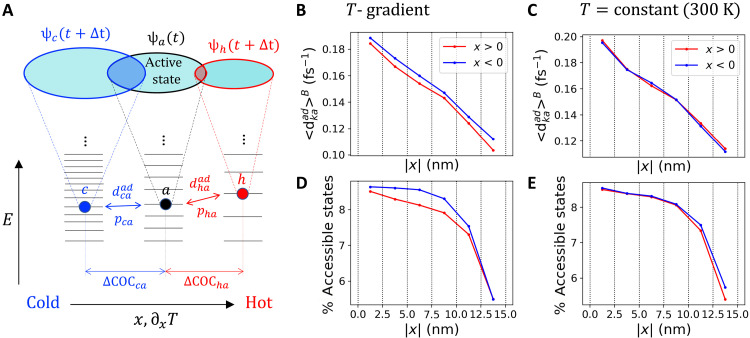
Interpretation of the net drift velocity from the hot to the cold side observed in FOB-SH simulations with a temperature gradient. (**A**) Energy levels are schematically shown for valence band (adiabatic) states with their COC located in the bin centered at 300 K (black) and in adjacent bins toward lower (blue) and higher temperature (red). The active valence band state ψ_a_ governing nuclear dynamics at time *t* and example adiabatic states ψ_c_ and ψ_h_ at time *t* + Δ*t*, which are a distance ΔCOC*_ca_* and ΔCOC*_ha_* displaced toward the cold and hot sides, respectively, are shown as ellipses, with increasing size from hot to cold to reflect their increasing IPR. NACEs ($d_{\mathrm{ka}}^{\text{ad}}$) and surface hopping probabilities (*p_ka_*) between ψ_a_ and ψ_c_ ($d_{\mathrm{ca}}^{\text{ad}}$, *p_ca_*) and between ψ_a_ and ψ_h_ ($d_{\mathrm{ha}}^{\text{ad}}$, *p_ha_*) are indicated. (**B** and **C**) Position-resolved thermally averaged NACE (Eq. 16) where $P_{k}=d_{\mathrm{ka}}^{\text{ad}}$, toward the cold side (data in blue) and hot side (data in red) for simulations under (B) a temperature gradient ∂*_x_T* = 2.8 K/nm and (C) at a constant temperature of 300 K. (**D** and **E**) Position-resolved percentage of thermally accessible states, *N*^acc^(*x_i_*)/∑*_j_N*^acc^(*x_j_*) × 100% where *N*^acc^(*x_i_*) is given by Eq. 15, toward the cold side (data in blue) and hot side (data in red) for simulations under (D) a temperature gradient ∂*_x_T* = 2.8 K/nm and (E) at a constant temperature of 300 K.

What causes the directional motion of the hole wave function from hot to cold? Our mechanistic proposal based on FOB-SH simulations is illustrated in Fig. 5A. We assume that the hole wave function ψ_a_ at a given time *t* is located in the central bin (shown schematically as an ellipse in the middle of Fig. 5A), and we analyze the likelihood for transitions from ψ_a_ to one of the states toward the cold side (denoted “cold states”, e.g., ψ_c_ indicated by an ellipse to the left) or to one of the states toward the hot side (denoted “hot states”, e.g., ψ_h_ indicated by an ellipse to the right). In other words, we aim to explain why, on average, ψ_a_ is more likely to transition to cold than to hot states, thereby generating the negative drift velocity 〈*v_x_*〉 obtained in the simulations. We find that major displacements of the hole wave function contributing to drift velocity are typically induced by thermally induced electronic transitions (surface hops) from ψ_a_ to other electronic states. The probability for transitions to occur is proportional to (i) the density of thermally accessible states, i.e., density of states that are within a few *k*_B_*T* of ψ_a_, and (ii) the magnitude of the nonadiabatic coupling between ψ_a_ and these states. We note in passing that the nonadiabatic coupling (or sometimes called derivative coupling) is the coupling between adiabatic or eigenstates of the electronic Hamiltonian and is related to but must be distinguished from the electronic coupling between the site-localized electronic states [see (*53*) for expressions relating these two quantities].

We find that the Boltzmann-averaged (i.e., thermally accessible) density of cold states (Fig. 5D, data in blue) is higher than for hot states (data in red), and this is the case for all distances between the COCs of ψ_a_ and the states on the cold or hot side (*x* = ΔCOC*_ka_*, *k* = c or h). Moreover, we find that the Boltzmann-averaged nonadiabatic coupling between ψ_a_ and the cold states is somewhat larger than between ψ_a_ and the hot states (Fig. 5B) and this is again the case for all distances *x*. Thus, according to this analysis of our simulation data, the negative drift velocity is due to the increasing density of thermally accessible states and the increasing nonadiabatic coupling between states with decreasing temperature. Both effects are a consequence of the decreasing electronic disorder, i.e., electronic coupling and site energy fluctuations, along the temperature gradient from hot to cold. In the control simulations at constant temperature, there is no such asymmetry with respect to states on the right-hand side (RHS) compared those on the left-hand side, as expected (see Fig. 5, C and E). We note that, in the central region of interest (∣*x*∣ < 10 nm), the number of accessible states varies only very weakly with respect to *x* (Fig. 5, D and E). The sharp drop for larger distances is due to the finite size of the simulation cell, which requires the number of states to tend to zero at the cell edge. The nonadiabatic coupling in Fig. 5 (B and C) is expected to decay with distance *x* for both the temperature gradient (Fig. 5B) and constant temperature case (Fig. 5C) because it is related to the overlap between two wave functions a distance *x* apart.

The asymmetries in the thermally accessible density of electronic states and nonadiabatic coupling with respect to states on the hot side and cold side are not unrelated. This is because the nonadiabatic coupling is inversely proportional to the energy difference between the coupled states, $d_{\mathrm{ja}}^{\text{ad}}\propto 1/\Delta E_{\mathrm{ja}}$ (*54*, *55*) (see Supplementary Note S11). We find good correlation between these two quantities (fig. S14, upper row). Thus, the larger density of energetically close cold states (small Δ*E_ja_*) compared to hot states contributes to the larger average nonadiabatic coupling for transition to cold states. Moreover, noting that the nonadiabatic coupling is given by $\langle \psi_{j}|{\dot{\psi }}_{a}\rangle$, an additional factor potentially further enhancing the average nonadiabatic coupling to cold states could be their greater delocalization compared to hot states. We find some correlation between nonadiabatic coupling and delocalization of electronic states (fig. S14, bottom row), although the scatter is relatively large and the correlation is not as strong as for the energy difference above.

### Seebeck coefficient

To link our simulations to the theory of thermoelectricity and the Seebeck coefficient, α, we consider the general expression for the current density along direction *x* (*J_x_*) in the presence of gradients of temperature (*T*), chemical potential (μ_c_), and electrical potential (ϕ) (*17*, *56*)

$$J_{x}=-\sigma \alpha \partial_{x}T-\frac{\sigma }{q}\partial_{x}\mu_{c}-\sigma \partial_{x}\phi$$ (1)

where σ is the electrical conductivity and *q* is the charge of the carriers, *q* = +1 for holes and −1 for electrons. (Note that α and σ are, in general, tensorial quantities but, for ease of notation, we omit indices.) The second and third terms on the RHS of Eq. 1 can alternatively be expressed in terms of the electrochemical potential $\bar{\mu }=\mu_{c}+q\phi$. The current density obtained from FOB-SH, *J_x_* = *qn*〈*v_x_*〉, where *n* formally corresponds to the concentration of the single charge carrier in our simulation cell, is the result of the first two terms on the RHS of Eq. 1. The first term, directly proportional to the Seebeck coefficient and the temperature gradient, drives the charge carrier from hot to cold, while the second term, proportional to the chemical potential gradient that is induced by the temperature gradient, drives the carriers in the opposite direction from cold to hot. The third term on the RHS of Eq. 1 is zero in the above simulations because no electrical potential gradients were applied. This differs from the usual experimental setup, where the electrical potential energy gradient, ∂*_x_*ϕ, is adjusted by an external electric field so that the net current arising from the first two contributions is compensated, i.e., *J_x_* = 0, and the resulting voltage is the open-circuit voltage, Δ*V*_oc_ = Δμ_c_/*q* + Δϕ.

Rearranging terms in Eq. 1, one finds

$$\alpha =-\frac{q}{e}\frac{\langle v_{x}\rangle }{\mu \partial_{x}T}-\frac{1}{q}\frac{\partial_{x}\mu_{c}}{\partial_{x}T}-\frac{\partial_{x}\phi }{\partial_{x}T}=\alpha_{v}+\alpha_{c}+\alpha_{e}$$ (2)

where μ = σ/(*en*) is the charge mobility along *x* and *e* is the elementary charge. Hence, the Seebeck coefficient is composed of three terms. The first term, α_v_, is proportional to the net current flow in the system. This contribution is of kinetic origin and depends on the charge transport mechanism. Notice that the factor *q*/*e* results in a different sign for the kinetic contribution for electrons and holes if they move in the same direction. The second and third terms, α_c_ and α_e_, are due to the gradient in chemical potential and external electric field, respectively; hence, they are of thermodynamic origin. We emphasize that the separation of the Seebeck coefficient in kinetic and thermodynamic contributions rigorously follows from Eq. 1 (*17*, *56*) without assuming any specific transport mechanism. Emin carried out a similar separation for systems that are in the phonon-assisted tunneling (hopping) regime by writing the total Seebeck coefficient in terms of a contribution of kinetic origin, α_transport_, and a contribution of thermodynamic origin, α_presence_ (*15*, *16*). Experiments are typically carried out under open-circuit conditions where the external electric field is adjusted such that the net current flow, and therefore the kinetic contribution α_v_, is equal to zero. Thus, in the experiment, the total Seebeck coefficient arises from the thermodynamic contributions only. Generally, the kinetic and thermodynamic contributions to the Seebeck coefficient will depend on the applied external electric field.

All three terms to the Seebeck coefficient (Eq. 2) can be obtained from computation. To evaluate the kinetic contribution (first term on the RHS of Eq. 2), we use the average drift velocity obtained from FOB-SH simulation under a temperature gradient without external field (Fig. 2A, data in magenta, 〈*v_x_*〉 = −0.89 nm ps^−1^ at *x* = 0, i.e., 300 K) and a hole mobility obtained from separate FOB-SH simulations at constant temperature without external field (μ = 33.0 cm^2^ V^−1^ s^−1^ at 300 K) using the same electronically active region size (50 × 7 unit cells) as in the simulations with a temperature gradient. (Notice that this value differs slightly from the one quoted above, 34.8 ± 3.5 cm^2^ V^−1^ s^−1^, which was obtained for a larger electronically active region.) This gives a kinetic contribution to the Seebeck coefficient α*_v_* = 97.4 ± 27.5 μV K^−1^ at 300 K. The thermodynamic contribution is given by α_c_ only because α_e_ = 0 at zero external field. α_c_ has previously been evaluated for the case of a nondegenerate semiconductor with parabolic bands, where a simple expression exists for μ_c_ (*52*, *57*). Such approximations should be regarded with caution in the case of rubrene, where the effect of thermal disorder on the band states is very strong. Here, we calculate $\frac{\partial \mu_{c}}{\partial T}$ explicitly by noting that the chemical potential μ_c_ is related to the free energy change upon insertion of a hole (electron) in the valence (conduction) band (Eqs. 18 and 19; see Materials and Methods and Supplementary Note S13 for details). At carrier density *n* = 2.74 × 10^15^ m^−2^, corresponding to a single carrier in the active region area of 50 × 7 unit cells, we obtain α_c_ = 331 ± 6 μV K^−1^. The total Seebeck coefficient at *T* = 300 K and *n* = 2.74 × 10^15^ m^−2^ is thus α = α_v_ + α_c_ = 429 ± 28 μV K^−1^.

We verify that the Seebeck coefficient computed from Eq. 2 is independent of the chosen external electric field by carrying out FOB-SH simulations subject to a temperature gradient but this time under open-circuit conditions, like in the experiment. To do so, we apply an external electric field in our simulation such that the average drift velocity and the resultant kinetic contribution to the Seebeck coefficient, α_v_, vanish. This should be the case for an external electric field of ≈3 × 10^3^ V cm^−1^ pointing in the direction from cold to hot. The results are shown in Fig. 3 (data in blue). The average drift velocity in the central bin is now much smaller, with a value of zero drift velocity falling within the statistical error bar, 〈*v_x_*〉 = 0.24 ± 0.33 nm ps^−1^ (panel A). The overall Seebeck coefficient, α = α_v_ + α_c_ + α_e_ = 397 ± 37 μV K^−1^, encompasses the value obtained without the field within the statistical uncertainty. Thus, the two simulation approaches (i.e., with and without an external electric field) for calculating the Seebeck coefficient within the framework of Eq. 2 yield consistent results. Note that the hole wave function is still very dynamic, frequently moving toward the cold or the hot side but at about equal amounts of time, thus averaging close to zero. We also find that the position-resolved IPR remains virtually unchanged when compared to the results without the electric field (Fig. 3C, magenta versus blue). This is expected because the above field strength corresponds to an electrostatic site energy difference between adjacent rubrene molecules along the *a* direction of only about 0.2 meV, which is much smaller than the electronic coupling (100 meV), thus has very little impact on the band structure and delocalization of valence band states.

### Comparison to the experiment

To validate our simulations, we carried out experimental measurements of the Seebeck coefficient in rubrene single crystals as a function of carrier concentration (*n*). These are measured under temperature differences of typically 5 to 10 K across a channel length of 420 μm and under open-circuit conditions (see Materials and Methods and Supplementary Note S14 for further details).

**Fig. 6.**
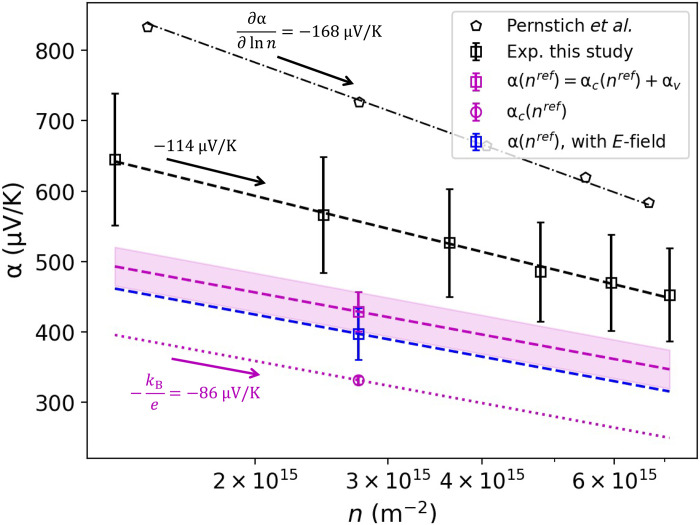
Experimental and calculated Seebeck coefficient α as a function of carrier concentration *n*. Black squares and pentagons correspond to experimental data from this study and (*58*), respectively, with best fits indicated with black dashed lines. The chemical potential contribution to the Seebeck coefficient, α_c_ (Eq. 20), computed at a reference carrier concentration *n*^ref^ = 2.7 × 10^15^ m^−2^, is indicated by the magenta dotted line, α_c_(*n*^ref^ = 2.7 × 10^15^m^−2^) = 331 ± 6 μV K^−1^. The total calculated Seebeck coefficient (for simulations with no external electric field, α_e_ = 0) is obtained by adding the kinetic contribution α_v_ = 97.4 ± 27.5 μV K^−1^, α(*n*^ref^) = α_c_(*n*^ref^) + α_v_ = 429 ± 28 μV K^−1^ (magenta dashed line). The shaded area indicates ± the SE in the calculation of α at *n*^ref^. For simulations using an external electric field, the total Seebeck coefficient is plotted with the blue dashed line.

Figure 6 shows the experimental Seebeck coefficients obtained in the present study (black squares) and those from (*58*) (black pentagons). The best fits of experimental data points to *A* + *B* ln (*n*), where *A* and *B* are optimization parameters, are indicated with black dashed lines. The total computed Seebeck coefficient, α = α_c_ + α_v_ + α_e_, obtained without and with an external electric field in FOB-SH simulations are indicated at *n* = 2.7 × 10^15^ m^−2^ corresponding to the carrier density present in FOB-SH simulations (squares in magenta and blue, respectively), along with α_c_ (circle in magenta). The concentration dependence of computed α and α_c_ (lines in magenta and blue) is given by Eq. 20. The uncertainty in α due to the statistical error from FOB-SH simulations is indicated over the entire concentration range by the magenta shaded region.

The computed total α = 429 ± 28 μV K^−1^ and α = 397 ± 37 μV K^−1^ obtained from FOB-SH simulations without and with external electric field, respectively, compares favorably with the experimental estimate at the same carrier density (*n* = 2.7 × 10^15^ m^−2^), 557 ± 82 μV K^−1^, with experimental and computational error bars nearly overlapping. The experimental slope ∂α/∂ ln (*n*) = −114.4 μV K^−1^ is also in reasonable agreement with the slope predicted by theory, ∂α/∂ ln (*n*) = −*k*_B_/*e* = −86.2 μV K^−1^. The remaining discrepancy could be caused by model assumptions, for instance, that the thermoelectric effect remains linear over the 1000-fold larger temperature interval applied in the simulations when compared with the experiment. Another source for the remaining discrepancy could be the presence of shallow trap states in experimental field-effect transistors (the existence of which is confirmed by observing the experimental threshold voltage shift as a function of temperature), which are known to cause an enhancement of the Seebeck coefficient and the slope ∂α/∂ ln (*n*) as well as a decrease in the experimental mobility (*3*). Therefore, the experimentally measured Seebeck coefficients are expected to be larger than the simulation where intrinsic valence band states are considered.

In Fig. 6, we also plot data taken from Pernstich *et al.* to highlight the sample-to-sample variation present in measurements (*58*). This data shows a substantially larger magnitude of the Seebeck coefficient and slope, ∂α/∂ ln (*n*) = −168 μV K^−1^. Experimental variations are likely due to differences in crystal quality, especially at the dielectric interface, as well as differences in the exact experimental setup and measurement methods. We highlight these differences to contextualize the comparison between the simulation and experimental data, where a similar magnitude of discrepancy is seen within different experimental determinations of the Seebeck coefficient. In addition, we note that, although it is generally believed that a two-dimensional (2D) model describes very well the charge transport situation in a field-effect transistor, the carriers in the experiment are not confined in the third dimension, which may also have a small effect on the charge carrier compared to the simulation. Even in a 2D model, transport is anisotropic so any misalignment of the high-mobility crystal axis with the temperature gradient will also result in some discrepancy.

## DISCUSSION

In this work, we have simulated the quantum dynamics of a charge carrier in an OS subject to a temperature gradient, providing a dynamical perspective and understanding of a transport problem that is typically approached from a purely static perspective. At the heart of our dynamical perspective of thermoelectricity is the gradient in the thermal disorder of electronic couplings and site energies that gives rise to gradients in the spatially resolved density of electronic eigenstates and their nonadiabatic coupling causing the carrier wave function to move from hot to cold. The explicit time-dependent carrier simulation presented here has also allowed us to understand the Seebeck coefficient in terms of a kinetic (α_v_) and thermodynamic contribution (α_c_ + α_e_). We have shown that simulations with no external electric field, where the carrier migrates from hot to cold, and those with a field to cancel such motion yield consistent Seebeck coefficients. While, in the current simulations at low carrier density, the Seebeck coefficient was dominated by the thermodynamic contribution, at high carrier density relevant in practical situations, the thermodynamic contribution will strongly decrease and the kinetic contribution is expected to become very important if not dominating. One aspect not considered here is the effect of static structural disorder (*59*) or chemical doping (*60*), which act as additional sources of carrier localization and may have a profound impact on the Seebeck coefficient. It is possible to include such effects into our methodology by building them into our molecular model, as done previously where we have studied the impact of static structural disorder on carrier transport at constant temperature (*61*).

The mechanistic insight we have obtained from our simulations opens up an avenue for the design of OSs with improved Seebeck coefficients. Previously, focus has been placed on the chemical potential contribution to the Seebeck coefficient, α_c_, sometimes referred to as the entropy of mixing. Increasing the entropy of the charge carrier in the valence or conduction band by increasing the density of thermally accessible valence band states will lead to an increase in α_c_. Yet, for ordered OSs where charge transport occurs in the TD regime, we propose an additional route aimed at increasing the kinetic contribution to the Seebeck coefficient, α_v_, or equivalently the electric field contribution, α_e_, compensating α_v_ under open-circuit conditions.

Recall that thermoelectric transport in OS is driven by the gradient in thermal electronic disorder and that a sensitive probe of the disorder is the average delocalization of the carrier wave function (IPR in Fig. 3C). Thus, we expect that systems with increasing sensitivity of thermal electronic disorder and, concurrently, charge carrier delocalization to changes in temperature will exhibit increasing values of α_v_. Hence, we assert that α_v_ ∝ −Δ*L_x_*/Δ*T*, where *L_x_* is the localization length of the charge carrier (related to IPR). Assuming the validity of transient localization theory $\left[\mu_{x}=\left(e/\left(k_{B}T\right)\right)L_{x}^{2}/\left(2\tau \right)\right]$ and temperature dependences of charge mobility μ*_x_* ∝ *T*^−*n*^ and localization time τ ∝ *T*^−*m*^, α_v_ ∝ −Δ*L_x_*/Δ*T* ∝ (*n* + *m* − 1). Hence, the kinetic contribution to the Seebeck coefficient is predicted to increase with increasing exponents of the temperature dependences of μ and τ. This hypothesis could be tested in the future work on a range of systems exhibiting varying temperature dependences of localization length or mobility.

## MATERIALS AND METHODS

### Fragment orbital–based surface hopping

FOB-SH is a mixed quantum-classical dynamics method based on fewest switches surface hopping, which allows for simulation of charge transport on the true nanoscale (10 to 100 nm) (*42*–*44*). In this method, a single excess charge carrier (in this work, hole carrier) is propagated in space and time according to the time-dependent Schrödinger equation under the influence of time-dependent classical nuclear motion. The single-particle time-dependent wave function, Ψ(*t*), is expanded in a basis of orthogonalized time-dependent frontier orbitals, which mediate charge transport

$$|\Psi \left(t\right)\rangle =\sum_{l=1}^{M}u_{l}\left(t\right)|\phi_{l}\left(R\left(t\right)\right)\rangle$$ (3)

where **R**(*t*) denotes time-dependent nuclear positions. In the case of hole (electron) transport, the basis functions ϕ*_l_* are the orthogonalized HOMOs [LUMOs (lowest unoccupied molecular orbitals)] of the molecules, which constitute the crystal lattice. The valence band in which the excess hole is propagated is described by the following Hamiltonian

$$H=\sum_{k}\epsilon_{k}|\phi_{k}\rangle \langle \phi_{k}|+\sum_{k\neq l}H_{\mathrm{kl}}|\phi_{k}\rangle \langle \phi_{l}|$$ (4)

where ϵ*_k_* = 〈ϕ*_k_*∣*H*∣ϕ*_k_*〉 are the site energies, i.e., the potential energy of the system when the excess charge is localized on molecule *k*, and *H_kl_* = 〈ϕ*_k_*∣*H*∣ϕ*_l_*〉 are the electronic couplings. To facilitate simulations on large systems over long timescales, we have developed a parameterized approach that avoids explicit electronic structure calculations. Site energies ϵ*_k_* are calculated using a classical force field where molecule *k* is charged and all other molecules are neutral. Electronic couplings *H_kl_* are calculated using the efficient AOM (*45*, *46*), which assumes a linear relationship between electronic coupling and orbital overlap $H_{\mathrm{kl}}={\bar{C}\bar{S}}_{\mathrm{kl}}$, where ${\bar{S}}_{\mathrm{kl}}$ is the overlap between fragment orbitals *k* and *l* projected onto a minimal Slater-type orbital basis and $\bar{C}$ is a fitting parameter obtained by correlating ${\bar{S}}_{\mathrm{kl}}$ with reference *H_kl_* computed from density functional theory (DFT), specifically the POD method (*48*). We note that, for rubrene, this provides a very good approximation, with mean absolute errors (mean relative unsigned errors) of 5.8 meV (5.5%) and 3.2 meV (23.6%) for *a* and *b* direction AOM couplings with respect to DFT, *J_a_* and *J_b_*, respectively (*62*). Inserting Eq. 3 into the time-dependent Schrödinger equation yields

$$i\hbar {\dot{u}}_{k}\left(t\right)=\sum_{l=1}^{M}u_{l}\left(t\right)\left(H_{\mathrm{kl}}\left(R\left(t\right)\right)-i\hbar d_{\mathrm{kl}}\left(R\left(t\right)\right)\right)$$ (5)

where $d_{\mathrm{kl}}=\langle \phi_{k}|{\dot{\phi }}_{l}\rangle$ are the nonadiabatic coupling elements (NACEs) in the quasidiabatic site basis, which are usually close to zero, in contrast to the NACEs in the adiabatic basis $d_{\mathrm{kl}}^{\text{ad}}=\langle \psi_{k}|{\dot{\psi }}_{l}\rangle$. At any given time, the nuclear dynamics is propagated classically on one of the adiabatic states that results from diagonalizing the electronic Hamiltonian. This adiabatic state is denoted the active surface, *E*_a_(**R**(*t*)). At each time step, the Tully surface hopping probability for a surface hop from state *a* to another adiabatic state *j*, *p_ja_*, is calculated (*38*)

$$p_{\mathrm{ja}}=-\frac{\text{Re}\left(c_{j}^{*}c_{a}d_{\mathrm{ja}}^{\text{ad}}\right)}{{|c_{a}|}^{2}}\Delta t$$ (6)

where *c_j_* and *c_a_* are the wave function expansion coefficients of adiabats *j* and *a*, respectively, and Δ*t* is the nuclear time step. The probability to remain on the active surface is given by *p_aa_* = 1 − ∑_*j*≠*a*_*p_ja_*. A random uniform number is drawn to stochastically decide whether to attempt hop to a different surface *j*. Energy conservation after a successful hop is enforced according to the standard procedure of rescaling the velocity components in the direction of the nonadiabatic coupling vector (NACV). If the nuclear kinetic energy is not sufficient to fulfill energy conservation, then the hop is rejected and the nuclear velocity components along the direction of the NACV are reversed (*43*, *53*). In addition to the standard prescription of surface hopping, three extensions to the original algorithm are required to ensure convergence of mobility with system size, detailed balance, and good internal consistency. These extensions include a decoherence correction, trivial crossing detection, and elimination of decoherence-induced spurious long-range charge transfer. We refer to (*43*) and (*53*) for a detailed discussion of these important additions to the method. A drawback of the surface hopping method is that nuclei are treated as classical particles, which means that certain nuclear quantum effects including zero-point energy and tunneling are not included. We do not think this is a major problem for the current system because these effects typically become important for systems characterized by high energetic barriers, i.e., in the small polaron hopping regime.

### FOB-SH simulations at constant temperature

Initial atomic positions for rubrene were taken from the Cambridge Crystallographic Data Centre structure with identifier QQQCIG01. Thermal expansion of the unit cell was accounted for by using temperature-dependent lattice parameters determined by a linear fit to the experimental temperature-dependent lattice parameters of (*49*) (see Supplementary Note S3 for details). The unit cell was optimized for each temperature under the constraint of fixed lattice parameters corresponding to that temperature. The force field used was based on the general Amber force field parameters, with selected parameters reoptimized to better reproduce results from ab initio MD simulations as reported in (*63*) (see Supplementary Note S1 for details). Supercells composed of 54 × 13 × 1 unit cells were prepared and equilibrated to 200, 225, and 250 K, and slightly smaller supercells composed of 50 × 13 × 1 unit cells were equilibrated to 275, 300, 325, and 350 K for 200 ps in the NVT ensemble applying periodic boundary conditions, a Nosé-Hoover thermostat, and an MD time step of 1 fs. This was followed by at least 325-ps MD simulation in the NVE ensemble. Initial positions and velocities for the swarm of FOB-SH trajectories were drawn from snapshots separated by 0.5 ps from the equilibrated NVE trajectory. For each trajectory, the wave function was initialized on a single molecular site (i.e., diabatic state) located at the corner of the simulation box and propagated for 900 fs in the NVE ensemble using an MD time step of 0.05 fs and an electronic time step of 0.01 fs for integration of Eq. 5 using the Runge-Kutta algorithm to fourth order. At least 650 FOB-SH trajectories were run for each temperature. The initial 200 fs of dynamics, corresponding to quantum relaxation from the initial diabatic state, was neglected in all analysis. The diffusion tensor for each temperature was obtained from a linear best fit of the MSD against time (Eq. 9), between 200 and 900 fs (see fig. S5). Mobility was calculated using the Einstein relation (Eq. 10). The mobility values reported herein are well converged with system size, even for the low temperatures where hole delocalization is extensive. A detailed analysis of the convergence is given in Supplementary Note S4 and tables S4 and S5.

### FOB-SH simulations with a temperature gradient

A supercell of size 120 × 7 × 1 (using the 300-K lattice parameters) was defined in periodic boundary conditions. A saw-tooth temperature profile was achieved by defining two thermal bath regions of size 10 × 7 × 1 unit cells at temperatures of 250 and 350 K, separated by 50 unit cells along the *a* direction. The temperature in the thermal bath regions was constrained to the target temperatures through a velocity rescaling procedure, as implemented in CP2K (*64*) (see Supplementary Note S9 for the detailed procedure). A set of 400 positions and velocities sampled every 0.5 ps from a 200-ps nonequilibrium run under the temperature gradient were used as initial coordinates for subsequent FOB-SH runs. The (nonperiodic) electronically active region in FOB-SH simulations was defined over one of the linear portions of the temperature profile (see fig. S8, top panel). To eliminate any possible boundary effects associated with initializing the wave function at a given position, the hole wave function was initialized on a single molecule at five different initial positions, uniformly spaced along the *x* direction of the FOB-SH active region (at 250, 275, 300, 325, and 350 K). Overall, 2000 trajectories of length of 5 ps were run (400 × 5 initial wave function positions). In all production runs, velocity rescaling to the target temperature was only applied within the thermal bath regions. The dynamics in the electronically active region evolved according to the standard FOB-SH algorithm, i.e., Newtonian dynamics on one of the adiabatic potential energy surfaces and rescaling of the velocity component parallel to the NACV after successful surface hops. The settings and integration time steps were the same as described above for FOB-SH simulation at constant temperature. Further simulation details are presented in Supplementary Note S9.

### FOB-SH simulations with a temperature gradient and an external electric field

The above FOB-SH simulations with a temperature gradient were repeated under the presence of a constant external electric field along *x* (*E_x_*). The effect of the field was simply modeled by a linear change of the site energies of the electronic Hamiltonian (Eq. 4), ϵ*_k_*(**R**) → ϵ*_k_*(**R**) − *qE_x_x_k_*, where *q* = +*e*, *x_k_* is the center of mass of molecule *k*, and *E_x_* = −∂*_x_*ϕ = 2.557 × 10^3^ V cm^−1^. The corresponding additional forces on the nuclei have been accounted for but are negligibly small for the small fields applied. The same simulation protocol was followed as for the simulations without external field except that trajectories were run to 3 ps rather than 5 ps due to our finding that trajectories of length of 3 ps are sufficiently converged (see fig. S15). All simulations were carried out with our in-house implementation of FOB-SH in the CP2K simulation package (*64*).

### Calculation of charge mobility and IPR

Hole mobilities (Fig. 2, C and D) were obtained from FOB-SH trajectories run at constant temperature. The MSD of the hole wave function is calculated as follows

$${\text{MSD}}_{\alpha \beta }=\frac{1}{N_{\text{traj}}}\sum_{n=1}^{N_{\text{traj}}}\langle \Psi_{n}\left(t\right)|\left(\alpha -\alpha_{0,n}\right)\left(\beta -\beta_{0,n}\right)|\Psi_{n}\left(t\right)\rangle$$ (7)

$$\approx \frac{1}{N_{\text{traj}}}\sum_{n=1}^{N_{\text{traj}}}\left(\sum_{k=1}^{M}{|u_{k,n}\left(t\right)|}^{2}\left(\alpha_{k,n}\left(t\right)-\alpha_{0,n}\right)\left(\beta_{k,n}\left(t\right)-\beta_{0,n}\right)\right)$$ (8)

where Ψ*_n_*(*t*) is the hole wave function in FOB-SH trajectory *n*, α = *x*, *y*, β = *x*, *y* are Cartesian coordinates along the crystallographic directions *a*, *b*, α_0,*n*_(β_0,*n*_) are the initial positions of the COC in trajectory *n*, α_0,*n*_ = 〈Ψ*_n_*(0)∣α∣Ψ*_n_*(0)〉, and *N*_traj_ is the number of FOB-SH trajectories. In Eq. 8, the coordinates of the hole are discretized and replaced by the center of mass of molecule *k* in trajectory *n*, α_*k*,*n*_, and α_0,*n*_ = 〈α*_n_*〉(0), where 〈α*_n_*〉(*t*) is the α coordinate of the COC at time *t* in trajectory *n*, $\langle \alpha_{n}\rangle \left(t\right)=\sum_{k=1}^{M}{|u_{k,n}\left(t\right)|}^{2}\alpha_{k,n}\left(t\right)$, and ∣*u*_*k*,*n*_(*t*)∣^2^ is the hole population of site *k* in trajectory *n*. The diffusion tensor *D*_αβ_ is given by half of the slope of MSD_αβ_

$$D_{\alpha \beta }=\frac{1}{2}\underset{t\to \infty }{\text{lim}}\frac{{\text{dMSD}}_{\alpha \beta }\left(t\right)}{dt}$$ (9)

which allows the charge mobility μ_αβ_ to be calculated from the Einstein relation

$$\mu_{\alpha \beta }=\frac{{\mathrm{eD}}_{\alpha \beta }}{k_{B}T}$$ (10)

where *e* is the elementary charge, *k*_B_ is the Boltzmann constant, and *T* is the temperature. Delocalization of the hole wave function Ψ(*t*) (Fig. 2A) is quantified using the IPR

$$\text{IPR}\left(t\right)=\frac{1}{N_{\text{traj}}}\sum_{n=1}^{N_{\text{traj}}}\frac{1}{\sum_{k=1}^{M}{|u_{k,n}\left(t\right)|}^{4}}$$ (11)

where *u*_*k*,*n*_(*t*) are the expansion coefficients of Ψ*_n_*(*t*) in the diabatic or site basis ϕ_*k*,*n*_ in trajectory *n* and *M* is the number of rubrene molecules in the electronically active region. The IPR of a given eigenstate or valence band state of the electronic Hamiltonian (Eq. 4), ψ*_i_* (Fig. 2B), is given by

$${\text{IPR}}_{i}\left(t\right)=\frac{1}{N_{\text{traj}}}\sum_{n=1}^{N_{\text{traj}}}\frac{1}{\sum_{k=1}^{M}{|U_{\mathrm{ki}}\left(t\right)|}^{4}}$$ (12)

where *U_ki_*(*t*) are the expansion coefficients of eigenstate ψ*_i_* in the site basis ϕ*_k_*.

### Calculation of drift velocity, 〈*v_x_*〉

Position-resolved drift velocities along the *a* crystallographic direction (Fig. 3A) were obtained as follows. The COC of the hole wave function along *x* (crystallographic direction *a*)

$$\langle x_{n}\rangle \left(t\right)=\sum_{k=1}^{M}{|u_{k,n}\left(t\right)|}^{2}x_{k,n}\left(t\right)$$ (13)

was calculated for each FOB-SH trajectory *n* every 2 fs. The drift velocity was calculated from the finite difference time derivative of the COC, *v*_*x*,*n*_(*t*) = [〈*x_n_*〉(*t* + δt) − 〈*x_n_*〉(*t*)]/δt with δt = 2 fs. Position bins of length of 4 unit cells (2.9 nm) along the *x* direction were defined, and a given velocity was associated with the bin containing the COC at time *t*. The velocity at each time step over all trajectories was binned this way, and the data in each bin were averaged over, giving 〈*v_x_*〉. The same binning procedure was used for the probability density of COC (Fig. 3B), obtained by counting the number of occurrences of the COC in each bin, and for the IPR of the hole wave function (Eq. 11) (Fig. 3C). We note that using a smaller δt for the calculation of COC drift velocity makes little difference to the average drift velocity in the central bin compared to the statistical uncertainty. This was checked for the simulations at constant temperature and those using both a temperature gradient and an external electric field, where the charge carrier wave function was printed every 0.5 fs (i.e., four times more frequently than for simulations with a temperature gradient and no external electric field). For the constant temperature simulations, using δt = 0.5 fs results in a mean drift velocity in the central position bin of 〈*v_x_*〉 = 0.15 nm ps^−1^, whereas using δt = 2 fs results in 〈*v_x_*〉 = 0.10 ± 0.24 nm ps^−1^. For simulations with both a temperature gradient and an external electric field, using δt = 0.5 fs results in 〈*v_x_*〉 = 0.28 nm ps^−1^ compared to 〈*v_x_*〉 = 0.24 ± 0.33 using δt = 2 fs. In both cases, the values obtained using δt = 0.5 fs are well within the statistical uncertainty of the values computed using δt = 2 fs. Details concerning the distribution of drift velocities in the case of constant temperature and with temperature gradient are given in Supplementary Note S10 and fig. S11. Convergence of drift velocity with the number of trajectories and trajectory length is demonstrated in fig. S15.

### Analysis of thermoelectric motion

For the analysis shown in Fig. 5 (B and D), we used 800 FOB-SH trajectories of length of 2 ps with the temperature gradient applied. All configurations where a successful surface hop occurred and the COC of the active adiabatic state (index *a*), 〈*x_a_*〉, was located within the central 10 unit cells (7.2 nm) along the *a* direction were included [$\langle x_{a}\rangle =\sum_{l=1}^{M}{|U_{l,a}\left(t\right)|}^{2}x_{l}\left(t\right)$ with *x_l_* the center of mass of molecule *l*]. This amounted to around 60,000 configurations overall. For each configuration, there are 700 adiabatic states ψ*_k_*, including the active state ψ*_a_*. Each adiabat *k* ≠ *a* was binned according to the difference in the COC position of that state and the COC position of the active state, ΔCOC*_ka_* = 〈*x_k_*〉 − 〈*x_a_*〉, using bin widths of 2.5 nm. The center of each bin is denoted *x_i_* in the following. To account for finite thermal accessibility of states *k* from state *a*, i.e., the fact that surface hops to adiabatic states deep inside the valence band may be energetically forbidden, a Boltzmann weight was assigned to each state according to its energy relative to the active adiabatic state energy, *E_k_* − *E_a_*

$$w_{k}^{B}=\text{min}\left[\text{exp}\left(\beta \left(E_{k}-E_{a}\right)\right),1\right]$$ (14)

Defining the number of thermally accessible states within a given bin *x_i_* as the sum of the Boltzmann weights of all states *k* within this bin

$$N^{\text{acc}}\left(x_{i}\right)=\sum_{k\in x_{i}}w_{k}^{B}$$ (15)

The thermally averaged property *P_k_* over adiabatic states in bin *x_i_*, $P_{k}=d_{\mathrm{ka}}^{\text{ad}},p_{\mathrm{ka}}$, IPR*_k_*, is given by

$${\langle P_{k}\rangle }^{B}\left(x_{i}\right)=\frac{\sum_{k\in x_{i}}P_{k}w_{k}^{B}}{N^{\text{acc}}\left(x_{i}\right)}$$ (16)

The NACE, Tully hopping probability, and IPR thermally averaged over adiabatic states in a distance bin *x_i_*, ${\langle d_{\mathrm{ka}}^{\text{ad}}\rangle }^{B}$, 〈IPR*_k_*〉^B^, and 〈*p_ka_*〉^B^ are shown for all distance bins in Fig. 5B and fig. S12 (A and C), respectively. The percentage of thermally accessible states in a distance bin *x_i_*, *N*^acc^(*x_i_*)/∑*_j_N*^acc^(*x_j_*) × 100%, is shown for all distance bins in Fig. 5D. Moreover, the total thermally weighted probability for a surface hop to any state in bin *x_i_*, *N*^acc^(*x_i_*)〈*p_ka_*〉^B^(*x_i_*), is shown in fig. S12F. To facilitate a like-for-like comparison with the 300-K constant temperature simulation (Fig. 5, C and E, and fig. S13), the same procedure was carried out at a constant temperature of 300 K using an identical active region size of 50 × 7 unit cells and identical initial conditions.

### Chemical potential contribution to Seebeck coefficient, α_c_

The chemical potential of holes in the valence band is equal to the free energy change upon hole insertion into the band. It depends on hole density *n* and temperature *T*. For a given reference hole density, *n*^ref^, and *T* it is given by

$$\mu_{c}^{\text{ref}}\left(T,n^{\text{ref}}\right)=F_{\text{hole}}\left(T,n^{\text{ref}}\right)-F_{\text{neutral}}\left(T,n^{\text{ref}}\right)$$ (17)

$$=-k_{B}T\text{ln}{\langle \sum_{i}^{\text{vb}}e^{\beta [E_{i}\left(R\right)+E_{\text{neutral}}\left(R\right)}\rangle }_{E_{\text{neutral}}\left(R\right)}^{n^{\text{ref}}}$$ (18)

where *F* denotes the free energy, *E_i_*(**R**) is the *i*th valence band (vb) state at nuclear positions **R** [i.e., eigenstate of the electronic Hamiltonian (Eq. 4), where the index *i* runs from the top to the bottom of the valence band], and *E*_neutral_(**R**) is the energy of the neutral system at nuclear positions **R**. The brackets denote taking the ensemble average over configurations sampled from an MD trajectory of the neutral system at carrier density *n*^ref^. Note that *E_i_* are electronic energy levels, i.e., *E_i_* decreases with increasing hole excitation energy (see also Fig. 2B), thus the positive sign in the exponent of the Boltzmann weight. A derivation of Eq. 18 is presented in Supplementary Note S13. The chemical potential at a general carrier density *n* = 1/*A*, where *A* is the area of the electronically active region within the *a*-*b* plane, is given by

$$\mu_{c}\left(T,n\right)=\mu_{c}^{\text{ref}}\left(T,n^{\text{ref}}\right)+k_{B}T\text{ln}\frac{n}{n^{\text{ref}}}$$ (19)

The chemical potential contribution to the Seebeck coefficient, which is the second term on the RHS of Eq. 2, is then given by

$$\alpha_{c}=-\frac{1}{q}\frac{\partial \mu_{c}}{\partial T}=-\frac{1}{q}\left[\frac{\partial \mu_{c}^{\text{ref}}}{\partial T}+k_{B}\text{ln}\frac{n}{n^{\text{ref}}}\right]$$ (20)

The first term on the RHS of Eq. 20 can be obtained by calculating $\mu_{c}^{\text{ref}}\left(T,n^{\text{ref}}\right)$ at different temperatures around 300 K and taking the slope. Details on these calculations are presented in Supplementary Note S13 where we also show that the numerical results are virtually independent of the chosen reference concentration (*n*^ref^) (fig. S16 and table S8).

### Experimental details

Rubrene single crystals were grown via physical vapor transport under Ar flow from ≥98% pure rubrene powder (Sigma-Aldrich, used as purchased). The devices were made on 175-μm polyethylene terephthalate (Melinex ST504, DuPont Teijin Films). After sonic cleaning in acetone and isopropyl alcohol, the gate and heater electrodes were deposited via shadow mask with a 3-nm Cr adhesion layer followed by 20-nm Au. The gate dielectric is formed of a 500-nm layer of CYTOP, deposited by spin coating followed by thermal annealing at 90°C. The source and drain contacts (also 20-nm Au) were then evaporated, followed by manual placement of the grown single crystals aligning along the high-mobility direction.

The device architecture and the Seebeck measurement are carried out in a similar way as our previous study (*65*). All mobility and Seebeck measurements were performed in a Lake Shore CRX-4K cryogenic probe station using Keithley SMU models 2612B, 6430, and 2182 nanovoltmeters. For more details on the Seebeck measurement, see the Supplementary Materials.

## Supplementary Materials

### This PDF file includes:

Supplementary Notes S1 to S14 Figs. S1 to S21 Tables S1 to S8 References
