# Supplementary material for: Thermoelectric transport in molecular crystals driven by gradients of thermal electronic disorder
Source: Sci Adv. 2024 Oct 23;10(43):eadr1758. doi: 10.1126/sciadv.adr1758 (PMC11498209; doi:10.1126/sciadv.adr1758)
Supplement: Supplementary file 1 — Supplementary Notes S1 to S14 Figs. S1 to S21 Tables S1 to S8 References [file sciadv.adr1758_sm.pdf]

Science Advances
AAAS

Supplementary Materials for

Thermoelectric transport in molecular crystals driven by gradients of thermal electronic disorder

Jan Elsner *et al.*

Corresponding author: Jochen Blumberger, j.blumberger@ucl.ac.uk

Sci. Adv. **10**, eadr1758 (2024)
DOI: 10.1126/sciadv.adr1758

This PDF file includes:

Supplementary Notes S1 to S14
Figs. S1 to S21
Tables S1 to S8
References

1 Force field parameterization

FOB-SH simulations employ force fields for the calculation of diagonal Hamiltonian matrix elements (i.e. site energies). The matrix element H_{kk} corresponds to the energy of the system with molecule k charged and all other molecules neutral. The force field for the neutral system is based on the general AMBER force field (GAFF) (68) and parameters for the charged state were obtained by displacing the equilibrium bond lengths of the molecule with respect to the neutral state in order to reproduce the DFT reorganization energy, $\lambda = 0.152$ eV (see refs(35, 36) for details). The 300 K distributions of electronic couplings obtained using the force field for rubrene employed in previous work(35, 36) deviate slightly from those obtained using ab initio molecular dynamics with the optPBE-vdW density functional(63) (Prev. FF in Fig. 1 of the main text). This discrepancy stems from the use of an incorrect dihedral angle term linking phenyl side chains to the tetracene backbone such that parameters were set to match the dihedral terms describing in-plane interactions. This resulted in a slight misalignment of the phenyl side chains of optimized structures, visually apparent in Figure S1, which shows dimers extracted from optimized unit cells using the previously employed FF and optPBE-vdW DFT, as well as an experimental structure (CCDC database identifier QQQCIG01) (69). While this oversight has little effect on the conclusions drawn in previous studies, we have now updated the relevant dihedral terms so that the dihedral angles of optimized structures and the 300 K distributions of electronic couplings obtained from force field molecular dynamics better reproduce those obtained from ab-initio molecular dynamics.

The orientation of the phenyl side chains relative to the tetracene backbone can be quantified through the relevant dihedral angles, as indicated in Figure S1(d). For each phenyl side chain, there are 4 dihedral angles to consider: $\phi_1 = \phi$(ca1-cp1-cp2-ca3), $\phi_2 = \phi$(ca1-cp1-cp2-ca4), $\phi_3 = \phi$(ca2-cp1-cp2-ca3) and $\phi_4 = \phi$(ca2-cp1-cp2-ca4). The dihedral energy term has the form

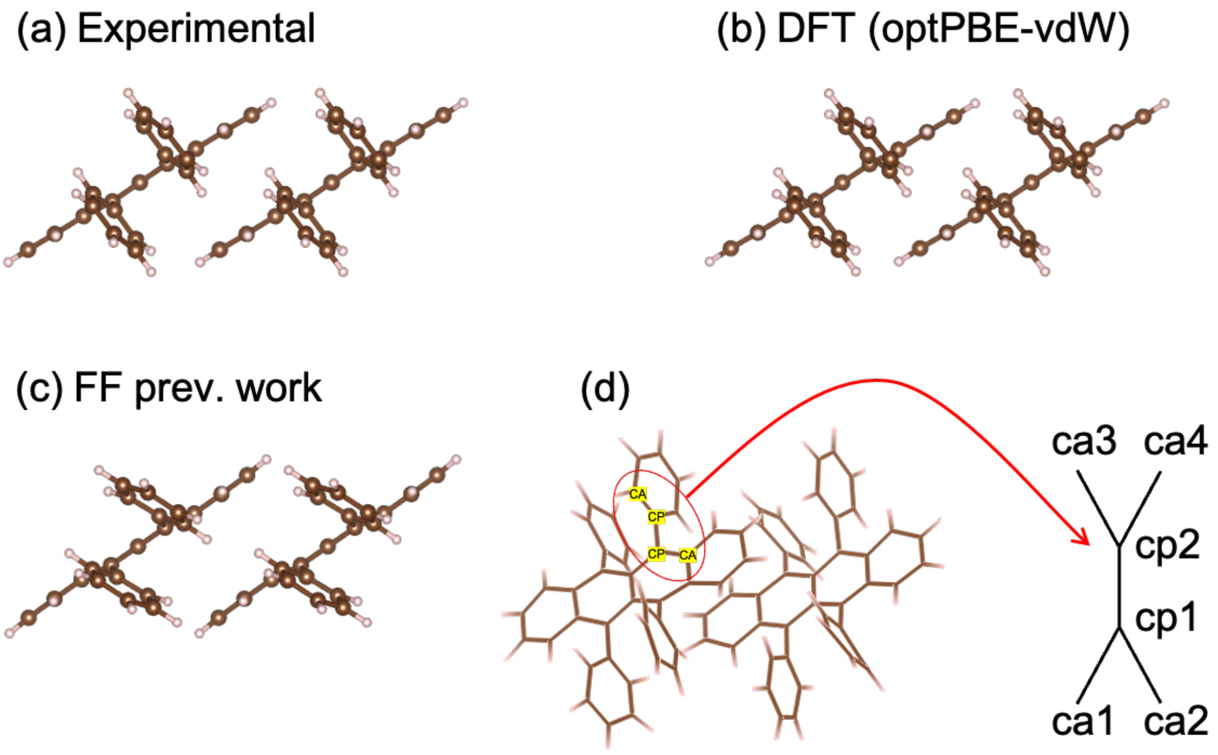

Figure S1: Rubrene dimer structures obtained from experiment, DFT and force-field optimization. (a) Experimental dimer (CCDC database identifier QQQCIG01(69)), (b) dimer extracted from a unit cell optimised with DFT using the optPBE-vdW density functional and (c) dimer extracted from a unit cell optimised using the previously employed force field. Optimisation using the latter results in a misorientation of phenyl side groups relative to the tetractene backbone, quantified by the pertinent connecting dihedral angles illustrated in (d).

$$E_\phi(\phi) = K_\phi(1 + \cos(2\phi - \phi_0)), \tag{S1}$$

where the parameters used previously are $K_\phi = 3.625$ kcal/mol/rad^2 and $\phi_0 = 180$ deg. This has an energy minimum at $\phi = 0$ deg, resulting in incorrect geometries for this system and an underestimation of the a direction electronic coupling, J_a. Table S1 lists dihedral angles ϕ_1, ϕ_2, ϕ_3 and ϕ_4, of the experimental structure and of 0 K optimised structures using DFT and force fields with different values of K_ϕ. Optimisation using the previously employed force field (Prev. FF in Table S1) yields dihedral angles that are either too large or too small. Setting $K_\phi = 0$ (i.e. turning off entirely) for dihedral terms linking the atoms depicted

in Figure S1(d) results in much better agreement with optPBE-vdW and the experimental structure (FF_0 in Table S1). Further improvement can be obtained by using a small value of $K_\phi = 0.6000$ kcal/mol/rad^2. We refer to this as FF_{opt} in Table S1, which is the force field we have used throughout the present study. We note that the parameters for all other dihedral angles (i.e. those which do not connect the tetracene backbone to the phenyl side chains) and all other interactions are the same as in previous work(35, 36).

Table S1: Dihedral angles for the experimental dimers and dimers extracted from optimised unit cells using potential energy surfaces.

	K_ϕ^a	ϕ_0^a	ϕ_1^b	ϕ_2^b	ϕ_3^b	ϕ_4^b
Experimental			75.9	111.8	96.6	75.7
optPBE-vdW			78.0	109.6	94.8	77.6
Prev. FF	3.625	180	49.5	129.1	116.4	65.0
FF_0	0.000	180	79.6	108.1	92.9	79.3
FF_{opt}	0.600	180	77.2	110.1	94.6	78.1

[a] Parameters for the dihedral energy term corresponding to atoms in Fig. S1(d), Eq. S1, for force field optimizations, in kcal/mol/rad^2.
[b] Dihedral angles for the atoms shown in Fig. S1(d), in degrees.

Table S2: Summary of electronic couplings and timescales of electronic coupling fluctuations using DFT and the different force fields described in the text.

	K_ϕ	J_a^a	$\langle J_a \rangle^b$	σ_a^b	J_b^a	$\langle J_b \rangle^b$	σ_b^b	τ_a^c	τ_b^c
optPBE-vdW/sPOD		110.2	107.1	28.2	-20.0	-17.0	7.7	78.5	96.3
Prev. FF/AOM	3.625	73.4	82.3	31.8	-18.5	-15.7	7.6	43.0	70.0
FF_0/AOM	0.000	108.8	111.1	31.0	-22.6	-16.0	9.0	64.8	79.9
FF_{opt}/AOM	0.6000	106.8	108.3	29.5	-21.9	-15.8	7.3	66.0	82.3

[a] Electronic couplings of dimers taken from optimized cells along the a and b crystallographic directions, J_a and J_b, respectively, in meV.
[b] Mean $\langle J_{a(b)} \rangle$ and root-mean-square fluctuations $\sigma_{a(b)}$ of the distribution of electronic couplings obtained by sampling dimers from a molecular dynamics trajectory at 300 K, in meV.
[c] Average timescale of coupling fluctuations, $\tau_{a(b)} = \int \mathrm{d}\omega\, \omega (S_{a(b)}(\omega)/\omega) / \int \mathrm{d}\omega (S_{a(b)}(\omega)/\omega)$, in cm^{-1}.

Electronic couplings of 0K optimized structures, as well as mean and root-mean-square fluctuations of the 300 K distributions, using DFT and the various force fields are listed in

Table S2. The distributions of electronic couplings were obtained by sampling dimers every 50 fs from molecular dynamics trajectories of length 15 ps (timestep 1 fs) at 300 K. The force field used in previous work (Prev. FF) underestimates J_a for the optimized structure, as well as the mean value of the 300 K distribution, $\langle J_a \rangle$. This is remedied by reducing the force constant of the relevant dihedral terms. Best agreement with respect to ab initio molecular dynamics is obtained using $K_\phi = 0.6000$ kcal/mol/rad^2 (FF$_{\text{opt}}$). The timescales of electronic coupling fluctuations, τ_a and τ_b in Table S2, are also much improved using FF$_{\text{opt}}$; see also Figure 1 of the main text.

Electronic couplings for structures obtained from optPBE-vdW MD were calculated using the projector operator-based diabatization (POD) method (48) in combination with the PBE density functional and uniform scaling by 1.325 (sPOD) (70), as outlined in Ref. 63. For structures obtained from force field MD, the analytic overlap method (AOM) (45, 46) was used, as employed in FOB-SH simulations.

2 Detailed description of results in Figure 1 main text

Figure 1(a) in the main text shows the distributions of electronic couplings J_a and J_b over trajectories at 300 K obtained with three different methods: ab-initio MD with the optPBE-vdW functional, classical MD using the force field employed in previous studies(35, 36) and classical MD using the optimized force field employed in this work, FFopt (SI section 1). In the first case, we utilise the DFT-based sPOD method for calculation of electronic couplings(48), whereas in the latter two cases we utilise the analytic overlap method (AOM), as employed in FOB-SH (46). We note significantly better agreement in the distributions of electronic couplings when using the optimized force field FFopt compared to the force field used previously (see SI section 1).

Figure 1(b) shows the normalised density of states (DOS) obtained using the different methods. The DFT/PBE density of states of the optimized structure is shown in the black dashed line. We note that the bandwidth has been scaled by a factor of 1.325 (hence the label sDFT) due to a tendency for the PBE functional to underestimate electronic couplings (70). The energy of the top of the valence band was set to 0. All other lines are obtained using the valence band Hamiltonian (Eq. 4 in the main text) with matrix elements sampled from the calculated distributions of electronic couplings. Dashed lines are for optimized structures with no electronic disorder i.e. all diagonal elements set to zero and the distributions of J_a and J_b are given by delta functions. Solid lines are for averages over 50 Hamiltonians with different realisations of disorder, obtained by sampling the 300 K distributions of electronic couplings. In each case, the peak of the DOS was aligned in energy with the peak of the sDFT/PBE DOS at -0.499 eV relative to the top of the valence band. We find that our valence band Hamiltonians achieve good accuracy compared to the full DFT DOS for the optimized structures, indicating that such valence band Hamiltonian accurately describes the valence band electronic structure (71). Excellent agreement is achieved with the FFopt Hamiltonians compared to the optPBE-vdW Hamiltonians (red vs cyan). The effect of finite temperature is to smear out density of states due to electronic disorder i.e. the spread in the

electronic couplings matrix elements, as indicated by the solid compared to dashed lines.

Figure 1(c) and (d) show the spectral density functions of the electronic coupling time series for J_a and J_b. The running integral of the spectral density yields the cumulative disorder including all frequencies up to ω, $\sigma_\alpha(\omega)$, allowing us to quantify the relative contribution of each mode, $\sigma_\alpha(\omega) = \left[\frac{8}{\beta\pi}\int_0^\omega d\omega' \, \frac{S_\alpha(\omega')}{\omega'}\right]^{\frac{1}{2}}$, where $S_\alpha(\omega)$ is the spectral density for time series J_α ($\alpha = a, b$) and $\beta = k_B T$. Including all frequencies $\omega \to \infty$ returns the root-mean-square fluctuation of the time series, which we note is similar using FFopt dynamics compared to optPBE-vdW dynamics (red vs cyan).

3 Temperature dependence of lattice parameters

All FOB-SH simulations were carried out at fixed cell volume. In the case of the constant temperature simulations over a range of temperatures, thermal expansion of the lattice was taken into account using a linear interpolation of the experimental temperature-dependent lattice parameters (49). The best fit line was extrapolated to yield lattice parameters for the higher temperatures (325 K and 350 K) not measured by experiment. For simulations under a temperature gradient from 250 K to 350 K centred at 300 K, linear expansion with temperature was assumed and the 300 K lattice parameters were used. Figure S2 shows the experimental lattice parameters along the a, b and c crystallographic directions (black lines), with linear fits in red dashed lines. The temperature-dependent lattice parameters employed in FOB-SH simulations are marked by red circles. Numerical values are listed in Table S3.

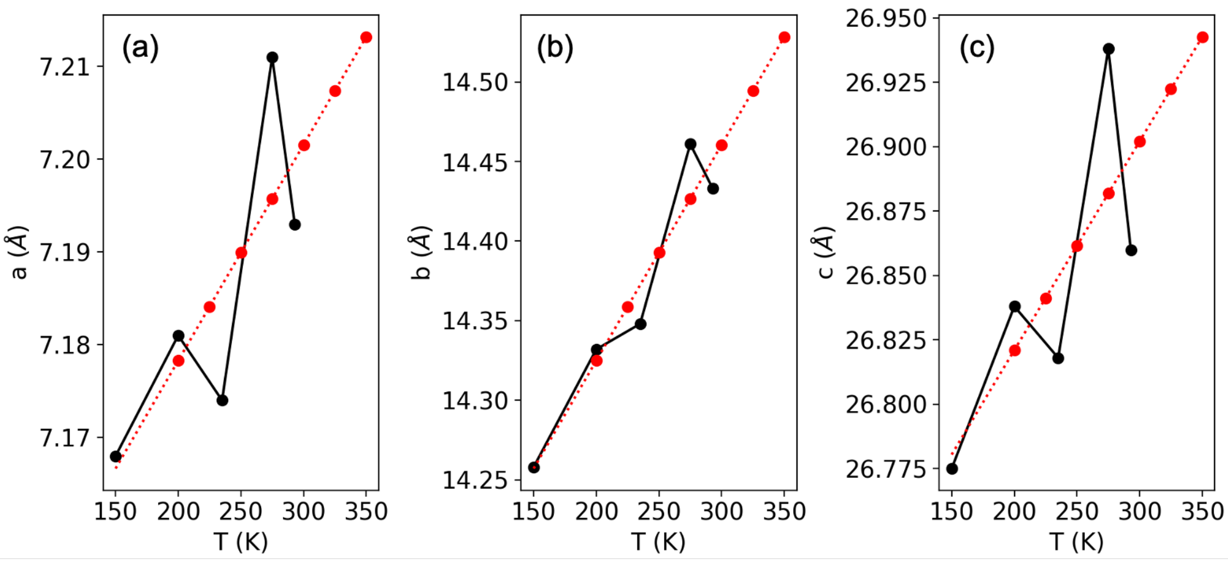

Figure S2: Experimental temperature-dependent lattice parameters of rubrene for the a, b and c directions from Ref. 49 in black. Best fit lines are shown in red (dashed) and values employed in FOB-SH simulations are marked by red circles.

Table S3: Temperature dependent lattice parameters of rubrene from experiment (49) and those employed in FOB-SH using a linear fit to the experimental values.

Temperature (K)		a (Å)	b (Å)	c (Å)
150	Exp	7.168	14.258	26.775
200	Exp	7.181	14.332	26.838
235	Exp	7.174	14.348	26.818
275	Exp	7.211	14.461	26.938
293	Exp	7.193	14.433	26.860
200	Fit	7.178	14.325	26.821
225	Fit	7.184	14.359	26.841
250	Fit	7.190	14.393	26.862
275	Fit	7.196	14.427	26.882
300	Fit	7.202	14.461	26.902
325	Fit	7.207	14.494	26.922
350	Fit	7.213	14.528	26.943

4 Convergence of hole mobility with respect to system size

Due to the increase in diffusivity with decreasing temperature, simulations at the lowest temperature, $T = 200$ K, are the hardest to converge with FOB-SH active region size. FOB-SH runs at different supercell sizes are required to ensure that mobility is converged with system size. If the cell is too small in a particular direction, the charge carrier wavefunction will be restricted in that direction once it reaches the vicinity of the boundary, leading to an underestimation in the slope of mean-squared-displacement with time. We can quantify the boundary effect by counting the fraction of trajectories where the wavefunction 'hits' the far boundary. A 'hit' refers to the center-of-charge of the wavefunction coming within some defined distance d_{hit} of the boundary. This is illustrated in Figure S3.

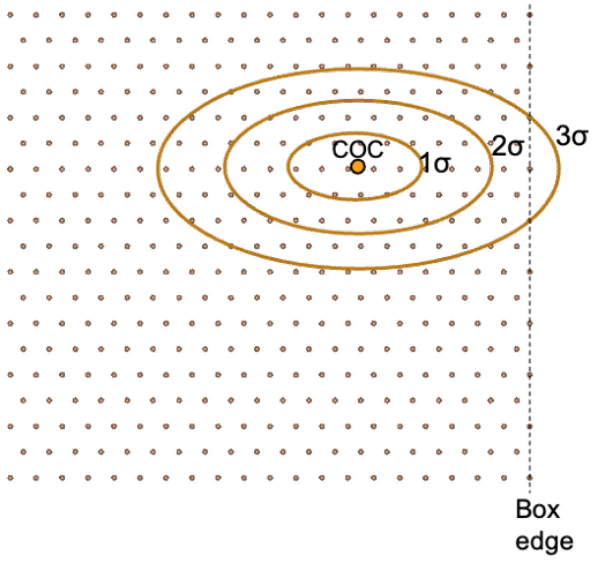

Figure S3: Illustration of how hits are counted. If the position of the centre-of-charge (COC) comes within $d_{\text{hit}} = n\sigma$ of the boundary, a hit is counted. The fraction of trajectories containing a hit gives an indication of whether the cell size is large enough. Too many hits indicate that the charge is significantly restricted by the boundary. In the present illustration a hit would be counted using $n \geq 3$.

We use two definitions of d_{hit}. In the first case, we define $d^{n}_{\text{av, x}}$ to be $n\times$ the standard deviation of the wavefunction projected along the x direction, averaged over all time steps:

$$d^n_{\mathrm{av},x} = n\bar{\sigma}_x = n\left\langle \sqrt{\langle x^2\rangle - \langle x\rangle^2} \right\rangle_t = n\left\langle \sqrt{\sum_k |u_k|^2 x_k^2 - \Big(\sum_k |u_k|^2 x_k\Big)^2} \right\rangle_t , \quad \text{(S2)}$$

where u_k are the wavefunction coefficients in the diabatic (site) basis, x_k denotes the position of site k along direction x and the average is taken over all time steps. An alternative choice is to define $d^n_{\mathrm{inst},x}(t)$ to be $n\times$ the instantaneous standard deviation of the wavefunction at time t, projected along the x direction.

$$d^n_{\mathrm{inst},x}(t) = n\sigma_x(t) = n\sqrt{\langle x(t)^2\rangle - \langle x(t)\rangle^2} = n\sqrt{\sum_k |u_k(t)|^2 x_k^2(t) - \Big(\sum_k |u_k(t)|^2 x_k(t)\Big)^2}. \quad \text{(S3)}$$

Equation S3 provides a more stringent criterion than equation S2 in the scenario where the wavefunction is located close to the boundary and undergoing a transient delocalisation event, $\sigma_x(t) \gg \bar{\sigma}_x$. In this case, a hit may be counted by equation S3 but not by equation S2.

The rubrene unit cell comprises four molecules (280 atoms) and spans two distinct high-mobility $a-b$ planes perpendicular to the out-of-plane c direction. The overall 3D periodic MD cell used in FOB-SH simulations includes both $a-b$ layers to ensure structural integrity, however the FOB-SH active region (where the FOB-SH electronic Hamiltonian is defined) includes only a single high-mobility layer, representing half the unit cell along the c direction. This is justified by the fact that electronic couplings along the c direction are orders of magnitude smaller than electronic couplings within the $a-b$ plane. In the following we denote supercell sizes by $N_a \times N_b$ and omit the dimension along the c-axis which is always 1/2 except where indicated otherwise.

For simulations at 200 K, we considered four supercell sizes with FOB-SH active regions of 32×18, 41×14, 50×13 and 54×13 unit cells. We note that while the overall MD cell is 3D periodic, the FOB-SH active region which defines the FOB-SH Hamiltonian is not. For simulations with the 32×18 active cell, a nuclear time step of 0.1 fs was used, whereas a smaller time step of 0.05 fs was used for all other cells. Such small nuclear time steps are

necessary to avoid trivial crossings (43). The percentage of hits within 900 fs along the a and b directions are listed in Table S4 and Table S5 using the definitions in equations S2 and S3, respectively, for different choices of n.

Table S4: Percentage of hits in the a and b directions for FOB-SH simulations of length 900 fs at 200 K using different active cell sizes. N_{traj} denotes the total number of FOB-SH trajectories. Hits are counted using $d^n_{\text{av},x}$ i.e. equation S2.

Active cell	Dimensions (nm × nm)	N_{traj}	% hits					
			$d^{n=1}_{\text{av},a}$	$d^{n=1}_{\text{av},b}$	$d^{n=2}_{\text{av},a}$	$d^{n=2}_{\text{av},b}$	$d^{n=3}_{\text{av},a}$	$d^{n=3}_{\text{av},b}$
32 × 18	22.98 × 25.80	199	3.0	0	20.6	0.5	33.2	0.5
41 × 14	29.44 × 20.06	396	2.5	0	6.6	0.5	11.9	0.8
50 × 13	35.91 × 18.63	419	1.0	1.4	3.3	3.6	5.5	5.3
54 × 13	38.78 × 18.63	687	0.6	0.4	1.9	1.5	2.9	3.6

Table S5: Percentage of hits in the a and b directions for FOB-SH simulations of length 900 fs at 200 K using different active cell sizes. N_{traj} denotes the total number of FOB-SH trajectories. Hits are counted using $d^n_{\text{inst},x}(t)$ i.e. equation S3.

Active cell	Dimensions (nm × nm)	N_{traj}	% hits					
			$d^{n=1}_{\text{inst},a}$	$d^{n=1}_{\text{inst},b}$	$d^{n=2}_{\text{inst},a}$	$d^{n=2}_{\text{inst},b}$	$d^{n=3}_{\text{inst},a}$	$d^{n=3}_{\text{inst},b}$
32 × 18	22.98 × 25.80	199	3.0	0	34.2	0.5	59.3	1.0
41 × 14	29.44 × 20.06	396	1.5	0	12.6	1.0	25.3	5.1
50 × 13	35.91 × 18.63	419	1.2	1.9	6.9	6.9	17.4	15.5
54 × 13	38.78 × 18.63	687	0.9	0.9	3.3	4.2	9.6	11.2

Simulations with the 32×18 cell result in many more hits along the a direction compared to the b direction, indicating that the anisotropy of the cell is not optimal and that the a direction length should be increased, while the b direction length may safely be reduced. This is ameliorated to some extent with the 41×14 cell, however there are still significantly more hits along a compared to along b. The 50×13 cell yields roughly the same number of hits in each direction, and this remains the case for the 54×13 cell, which further reduces the percentage of trajectories which hit the boundary. Such analysis is helpful for a sense of the optimal cell anisotropy, however convergence of the mean-squared-displacement with cell size must be checked explicitly.

Figure S4 shows the mean-squared-displacement along the a direction against time for the different cell sizes. Error bars are calculated by dividing the trajectories into 5 blocks,

and taking the standard deviation over the block averages. It is apparent from Figure S4(a) that cells which are smaller in the high mobility (a) direction result in smaller slopes for mean-squared-displacement with time, and hence smaller mobilities. The 50×13 cell is converged along the a direction with respect to the larger 54×13 cell. The 54×13 cell was used for temperatures between 200 K and 250 K. For higher temperatures, the 50×13 cell was used.

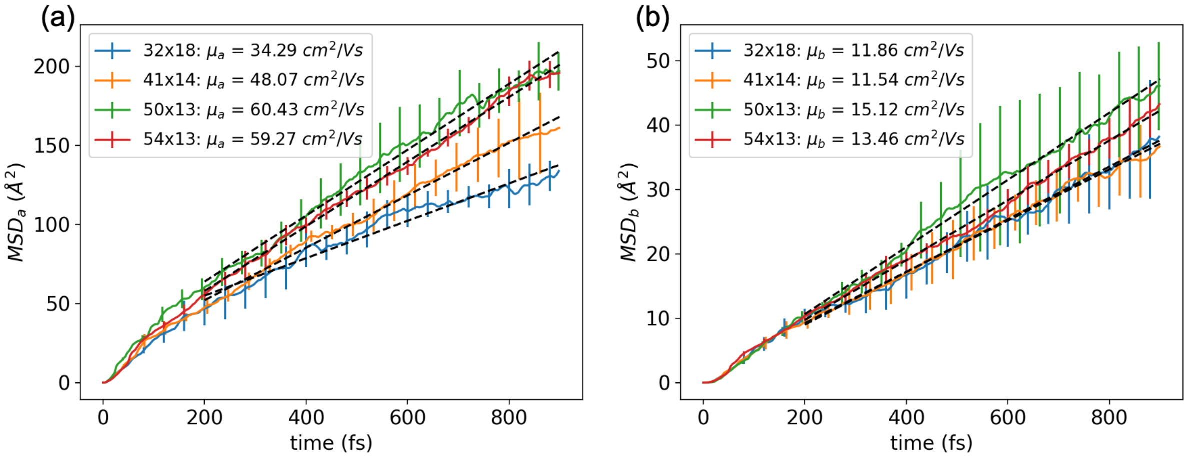

Figure S4: Mean-squared-displacement along (a) the a direction and (b) the b direction at 200 K against time for different active cell sizes. The good agreement for simulations with an active cell of 50×13 and 54×13 indicates that the supercell is converged.

5 Temperature dependence of MSD and hole mobility

Figure S5 shows converged plots of MSD against time for all constant temperature simulations. Error bars were calculated by partitioning the total number of trajectories into 5 blocks, and taking the standard deviation of the block averages. The first 200 fs were neglected in the fit, since the initial part of each trajectory corresponds to quantum relaxation from the initial diabatic state. The active region size, total number of trajectories and resulting values for mobility, are listed in Table S6.

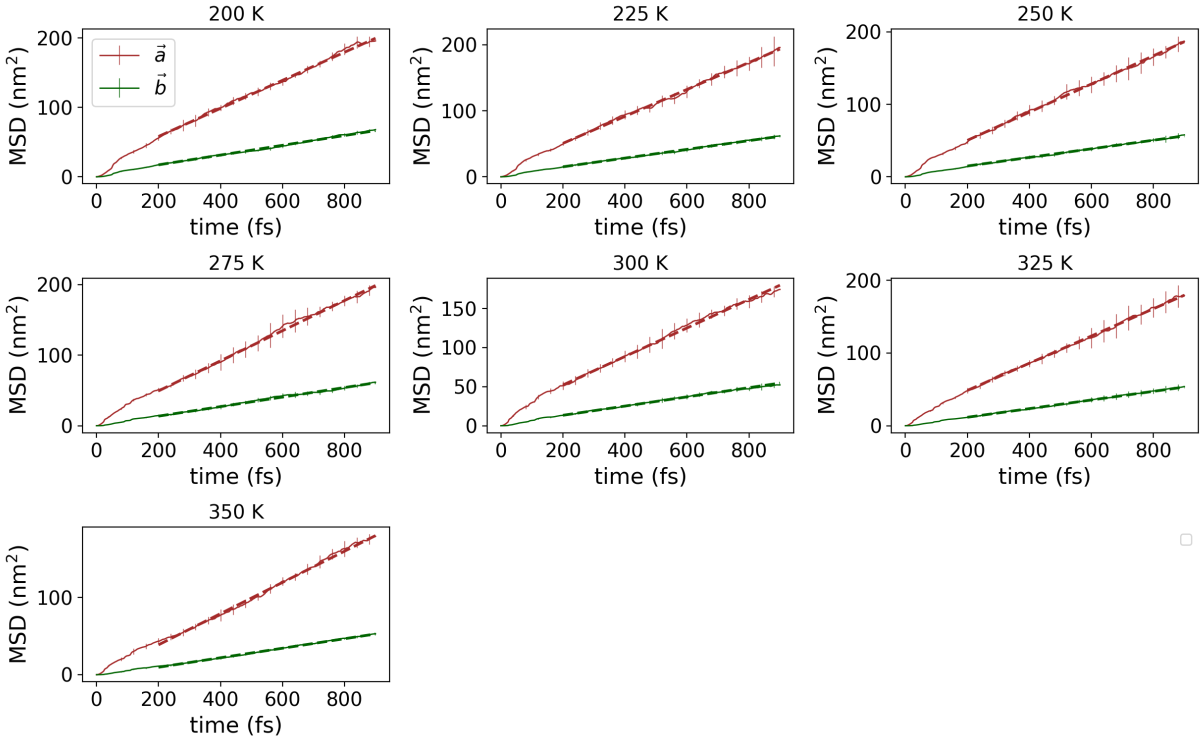

Figure S5: Mean-squared-displacement against time for all temperatures. Error bars indicate the standard deviation over 5 block averages. In all cases, MSD vs time is linear, indicating diffusive behaviour.

Table S6: FOB-SH mobilities and standard error along the a and b crystallographic directions of rubrene, for simulations at temperatures between 200 K and 350 K. The active region size, as well as number of trajectories is indicated.

Temperature (K)	Active region	N_{traj}	μ_a (cm$^2/Vs$)	μ_b (cm$^2/Vs$)
200	54×13	699	59.3 ± 13.1	13.2 ± 0.9
225	54×13	698	51.5 ± 4.6	12.0 ± 0.4
250	54×13	699	44.1 ± 5.6	8.4 ± 1.2
275	50×13	690	44.9 ± 3.9	8.5 ± 1.0
300	50×13	686	34.8 ± 3.5	6.5 ± 0.8
300	50×7	400	33.0 ± 5.1	
325	50×13	888	33.4 ± 2.5	6.8 ± 1.2
350	50×13	888	33.8 ± 5.2	6.5 ± 0.5

6 Temperature dependence of DOS, Valence band energy, IPR

The effect of increasing electronic disorder with temperature (see Table 1 of the main text) is to cause localisation of the eigenstates (i.e. adiabatic states) of the electronic Hamiltonian. Figure S6 shows the energy-resolved IPR of the valence band (also termed adiabatic) states, averaged over snapshots from 20 trajectories for each temperature. The heat-map axis indicates the number of states counted at a particular energy and IPR value. The energy of the valence band maximum was set to zero for each snapshot. The Boltzmann averages of the valence band energy and IPR are given by equations S4 and S5, respectively.

$$\langle E \rangle^B = \frac{\sum_k E_k \exp\{(E_k/k_B T)\}}{\sum_k \exp\{(E_k/k_B T)\}}, \tag{S4}$$

$$\langle \mathrm{IPR} \rangle^B = \frac{\sum_k \mathrm{IPR}_k \exp\{(E_k/k_B T)\}}{\sum_k \exp\{(E_k/k_B T)\}}, \tag{S5}$$

where k sums over the adiabatic states, k_B is the Boltzmann constant and T is temperature. These quantities are plotted as a function of temperature in panels S5(h) and S5(i).

In all cases, states are localised at the band edges and become increasingly delocalised towards the middle of the band. As temperature increases, the IPR of the adiabatic states at a given energy becomes smaller (most clearly illustrated in Figure 2(b) of the main text), indicating increasing localisation due to dynamic disorder. This can also be seen by considering the Boltzmann average of the IPR with temperature, shown in Figure S6(i). An additional effect of increased temperature is an increase in the Boltzmann averaged energy with temperature, shown in Figure S6(h). This is due to increased thermal energy with temperature, allowing the charge carrier to explore states deeper within the valence band where states are more delocalised. However, from panel S6(i), clearly the effect of increasingly localised states with temperature is more prominent and overall the states occupied by

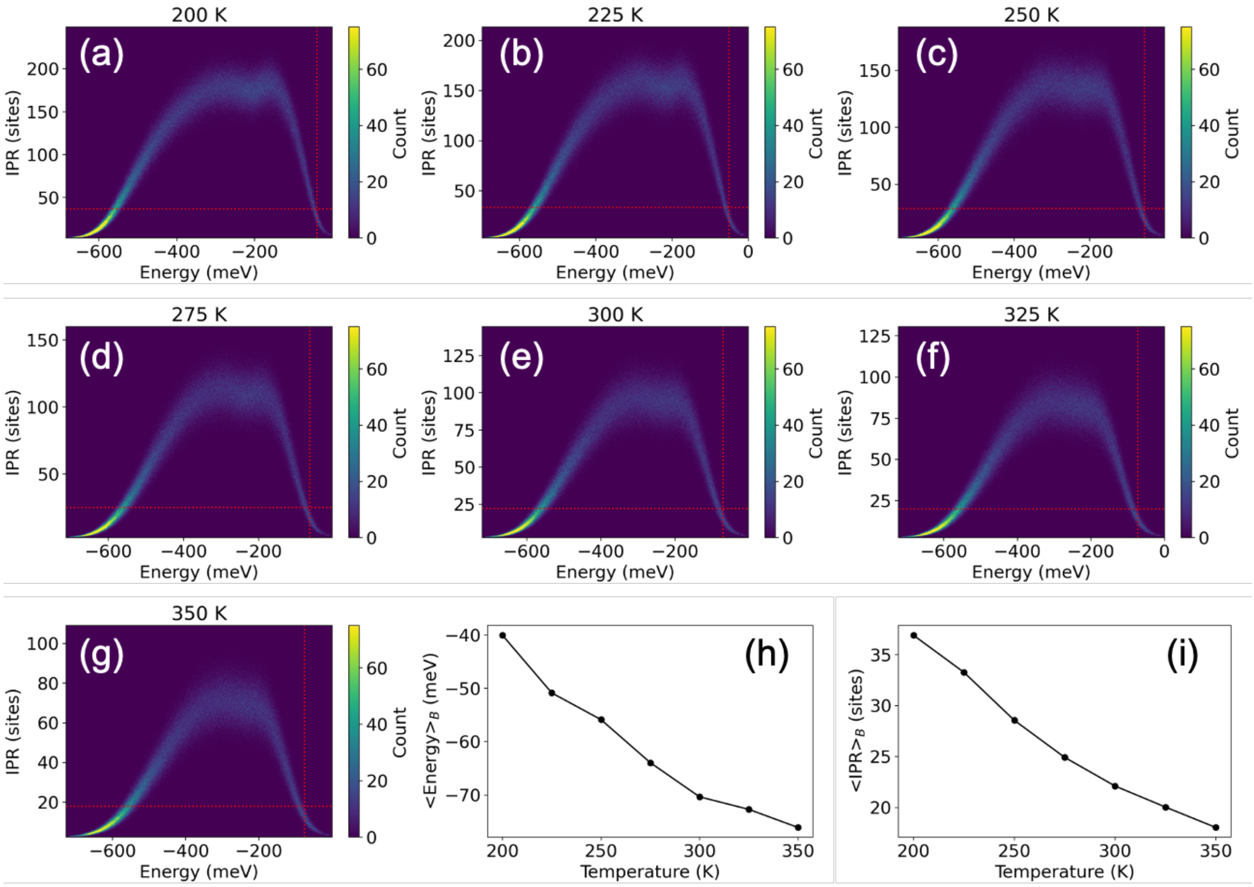

Figure S6: Energy and IPR-resolved density of valence band states of rubrene. (a)-(g) show 2D histograms of IPR against energy, where the heat-map axis indicates the number of states counted. Red dashed vertical and horizontal lines indicate the Boltzmann averaged energy and IPR, respectively. The quantities are plotted against temperature in panels (h) and (i).

a charge carrier will be more localised at higher temperatures(25).

7 Transient Delocalisation Mechanism for Constant Temperature Simulations

Figure S7(a), (b) and (c) show inverse participation ratio (IPR) of the charge carrier wavefunction as a function of time along a single FOB-SH trajectory at 300 K, 250 K and 200 K, respectively. The IPR exhibits significant fluctuations about the mean (indicated by the dashed line in magenta), which increases with decreasing temperature. Panels (d), (e) and (f) depict, respectively, the charge carrier wavefunction before, during and after an event of transient delocalization (TD). The charge carrier wavefunction is represented by superposing red ellipses on each molecular site (corresponding to fragment molecular orbital basis functions), with opaqueness proportional to the wavefunction site population $|u_k|^2$.

The TD mechanism, illustrated in Figure S7(d)–(f), allows for significant displacement of the charge carrier over distances up to the extent of the transiently delocalized state(36, 37). In the example shown, at $t = 418$ fs the charge carrier is delocalized over 5–6 molecules (IPR = 5.6) and nuclear dynamics is propagated on the ground state (i.e. top of the valence band, $E_a = 0$). Subsequently, a series of surface hops to excited valence band states take place such that the active adiabatic state at $t = 458$ fs is the 43rd valence band state, $E_a = -86.5$ meV. This is concomitant with TD of the wavefunction, which expands to cover approximately 39 molecules (IPR = 38.9) at $t = 458$ fs, a factor of 3 larger than the average value. Finally, following a series of surface hops back to the ground state, the wavefunction contracts and at $t = 470$ fs is momentarily delocalized over just 1–2 molecular sites (IPR = 1.8). Over the course of the TD event, the centre-of-charge of the wavefunction is displaced by approximately 3.5 nm. In general, displacements following a TD event may be larger or smaller and are limited only by the extent of the transiently delocalized state. Such TD events are driven by nonadiabatic transitions (i.e. surface hops) to excited valence band states, which become more delocalized towards the centre of the band (see Figure 2(b) of the main text). The extent of delocalization of the hole wavefunction in the TD state reflects

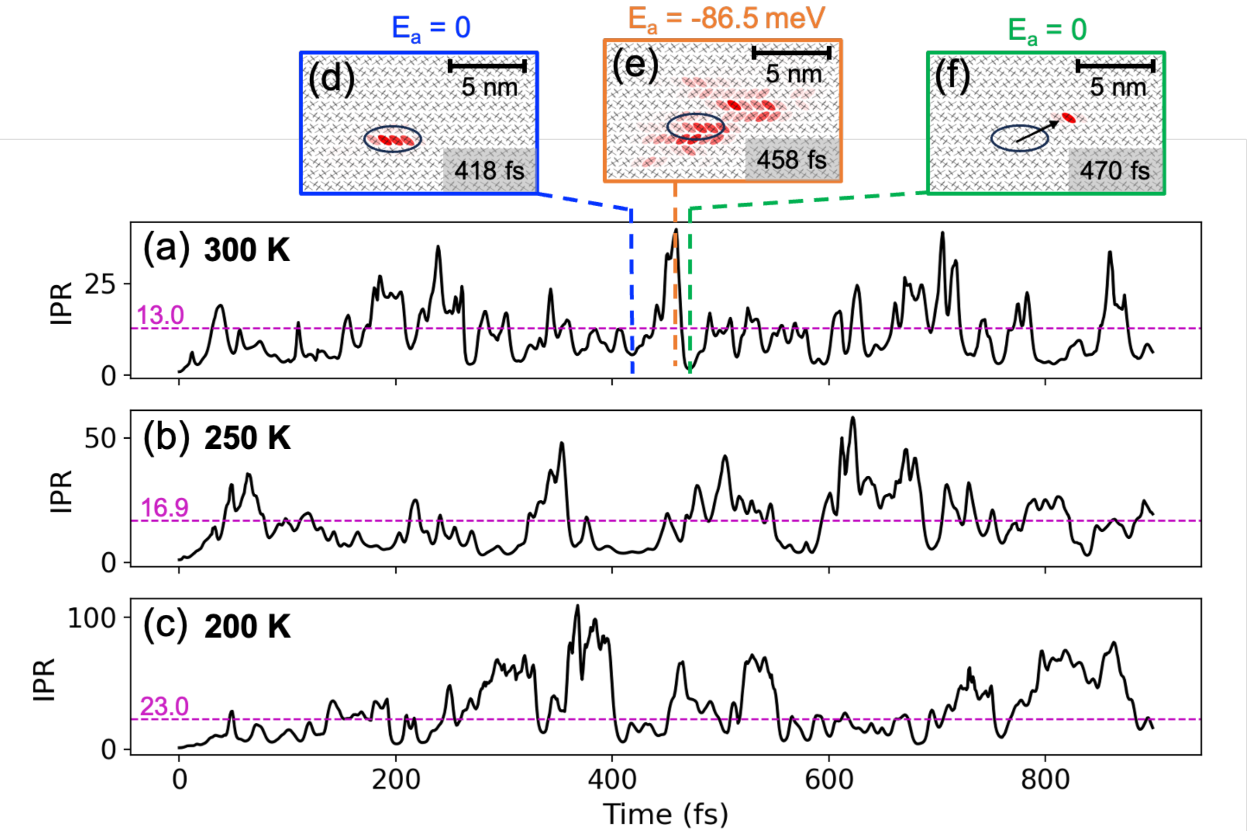

Figure S7: IPR of the charge carrier wavefunction (Eq. 11 of the main text) for individual trajectories at (a) 300 K, (b) 250 K and (c) 200 K. The average IPR (averaging over $t > 200$ fs and all trajectories) is indicated by the magenta dashed line in each case. Both the average and root-mean-square fluctuations of IPR become larger with decreasing temperature, see Table 1 of the main text. The mechanism of transient delocalisation (TD) is illustrated for the trajectory at 300 K in panels (d)–(f). The wavefunction is represented by superposing red ellipses onto each molecular site with opaqueness proportional to the site population, $|u_k|^2$. The energy of current active adiabatic state, E_a, is indicated in each case. Such transient expansions of the wavefunction are driven by surface hops to delocalized excited states (see Fig. 2(b) of the main text) and significantly contribute to the overall diffusivity(36, 37).

the extent of delocalization of the active adiabatic state on which the nuclear dynamics is propagated.

8 Transient Localization Theory Calculations

Transient localization theory (TLT) was used to calculate mobility for the different temperatures considered (Figure 2 of main text, magenta diamonds). The transient localization mobility is given by(19, 72)

$$\mu_{x(y)} = \frac{e}{k_B T} \frac{\bar{L^2}_{x(y)}(\tau)}{2\tau} \tag{S6}$$

where e is the elementary charge, k_B is the Boltzmann constant, T is temperature, τ is the timescale of lattice vibrations driving transient localization events, L is the so-called transient localization length and $\bar{L^2}$ denotes the average of L^2 over multiple realisations of disorder. The squared transient localization length is given by

$$L^2_{x(y)}(\tau) = \frac{1}{Z} \sum_{n,m} e^{\beta E_n} |\langle n|\hat{j}_{x(y)}|m\rangle|^2 \frac{2}{(\hbar/\tau)^2 + (E_m - E_n)^2} \tag{S7}$$

where Z is the partition function, $\hat{j}$ is the current operator and ($|n\rangle$, E_n) refer to the eigenstates and eigenvalues of a Hamiltonian corresponding to a particular realisation of disorder. Note, a positive sign is used in the Boltzmann factor since we consider hole transport. In practice, disordered Hamiltonians are constructed by sampling from specified distributions of electronic couplings for a given supercell size. The average squared localization length, $\bar{L^2}$ is obtained by averaging over the transient localization lengths corresponding to all Hamiltonians considered. Finally, mobility is calculated using Eq. S6. TLT mobility calculations for each temperature were carried out using freely available code (73) (https://github.com/CiuK1469/TransLoc, code version 0.4, downloaded 14th December 2020). We used a 39×26 supercell under periodic boundary conditions, which gave well-converged results in all cases, and we averaged over the transient localization lengths obtained from 50 distinct disordered Hamiltonians with matrix elements sampled from the temperature dependent distributions of electronic couplings listed in Table 1 of the main text. The timescale τ was calculated by averaging over the ab initio power spectrum for

J_a fluctuations, S_a (Fig. 1(c), cyan), $\tau = [\int \mathrm{d}\omega\, \omega(S_a(\omega)/\omega)/\int \mathrm{d}\omega(S_a(\omega)/\omega)]^{-1} = 0.43$ ps. We note that the dependence of mobility on τ in equation S6 is relatively weak due to the dependence of $L^2_{x(y)}$ on τ, compensating the denominator (73). Numerical values for TLT mobility at different temperatures are listed in Table S7.

Table S7: TLT mobilities along the a and b crystallographic directions for temperature between 200 K and 350 K.

Temperature (K)	μ_a (cm$^2/Vs$)	μ_b (cm$^2/Vs$)
200	62.5	16.4
225	55.1	13.9
250	50.3	12.4
275	41.4	9.9
300	39.5	9.1
325	35.0	7.9
350	31.1	6.8

9 Preparation of simulation cell with temperature gradient

Figure S8 shows the simulation box and temperature profile, averaged over 200 ps of MD. The supercell contains $120 \times 7 \times 1$ unit cells (4 molecules per unit cell) and is periodic in all dimensions. The temperature gradient is along x which is parallel to the a crystallographic direction. Since the overall cell is 3D periodic, the temperature at either side of the box along x must be the same, therefore a saw-tooth temperature profile is required. The FOB-SH active region, which is not periodic, is defined over one of the linear portions of the temperature profile ($50 \times 7 \times 1/2$ unit cells), as indicated.

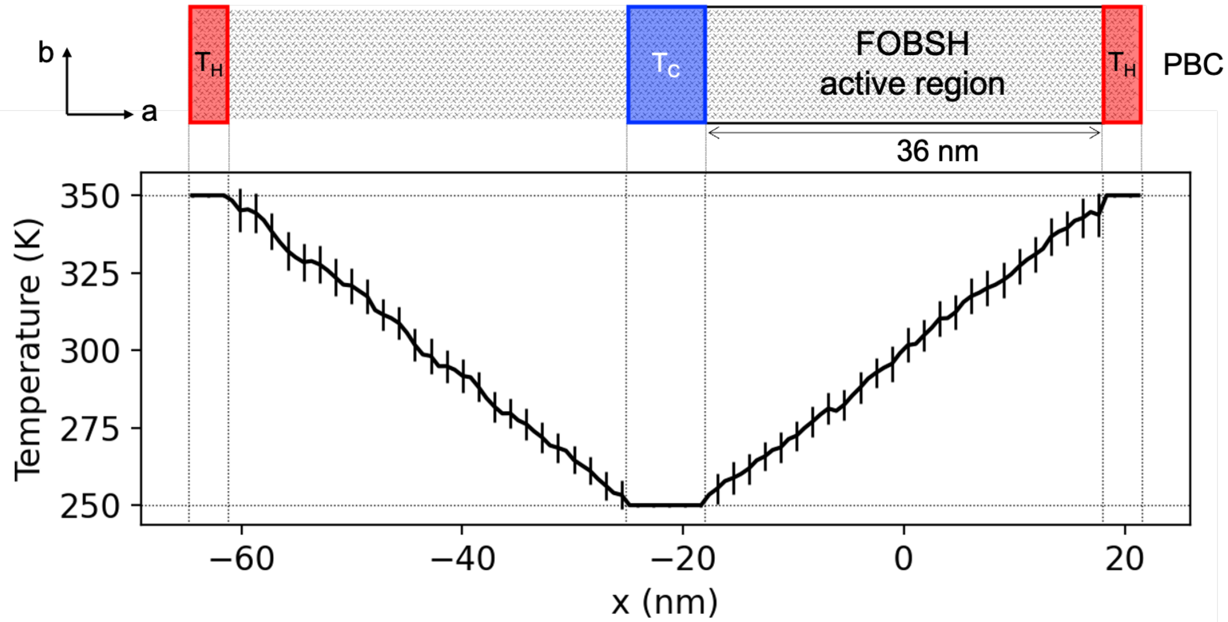

Figure S8: Full supercell for simulations under a temperature gradient and temperature profile, averaged over 200 ps of MD. Error bars represent the root-mean-square fluctuations in local kinetic temperature including all atoms within a slab of dimensions $1 \times 7 \times 1$ unit cells. Thermal bath regions, of size $10 \times 7 \times 1$ unit cells, are maintained at temperatures $T_C = 250$ K and $T_H = 350$ K through a velocity rescaling procedure. The thermal bath regions are separated by an active region (later used for hole propagation in FOB-SH), where the temperature varies with a uniform gradient. The supercell shown is periodically replicated in 3D and the active region in FOB-SH simulations (50×7 unit cells in the $a - b$ plane, 36.01 nm $\times$ 10.12 nm) is defined over one of the linear parts of the temperature profile, as indicated, and is not periodically replicated.

The temperature profile is achieved by defining thermal bath regions at 250 K and 350 K ($10 \times 7 \times 1$ unit cells, 280 molecules, 19600 atoms), as indicated in Figure S8, which are pinned to their respective temperatures through a velocity rescaling procedure. Velocity rescaling to the target temperature occurs whenever the difference between the instantaneous kinetic temperature, T_{inst}, and the target temperature, T_{target}, exceeds some defined tolerance, $|T_{\text{inst}} - T_{\text{target}}| > T_{\text{tol}}$. T_{tol} was set to 1 K in the bath regions. Figure S9(a) shows the temperature profile, averaged over 1 ps, after 50 ps of MD. The temperature profile is highly non-linear, indicating that a long time scale is needed for relaxation to the steady state using this approach. To speed up convergence of the temperature profile, we initially apply velocity rescaling over the full simulation cell. To do so we defined in the active region consecutive slabs with dimensions $1 \times 7 \times 1$ unit cells (28 molecules, 1960 atoms) and set the target temperature in each slab to the temperature expected from a linear temperature gradient. The temperature tolerance in the slabs was set to $T_{\text{tol}} = \{1, 5, 20\}$ K for sequential molecular dynamics runs of length 2 ps whilst a tolerance of $T_{\text{tol}} = 1$ K was applied to the thermal bath regions throughout the relaxation procedure. Hence, thermostatting of the active region was sequentially made weaker. Following this initial relaxation, thermostatting in the active region was turned off entirely keeping only the thermal bath regions thermostatted. The temperature profile achieved in this manner is linear and stable over long time scales ($\gg$ 200 ps, the time scale averaged over in Fig. S8), see Figure S9(b) where the profile was averaged over 1 ps, after 50 ps of MD. The FOB-SH simulations with temperature gradient were initialized from snapshots taken from this trajectory keeping only the thermal bath regions (and not the active region) thermostatted by velocity rescaling.

The root-mean-square fluctuations in temperature within a given slab (i.e. error bars in Fig. S8) are of the order 5 K, which determines the local temperature resolution in our simulations. The magnitude of temperature fluctuations shows a slight linear increase with local temperature, see Figure S10. This is consistent with the expected temperature dependence of temperature fluctuations in the NVT ensemble from statistical mechanics,

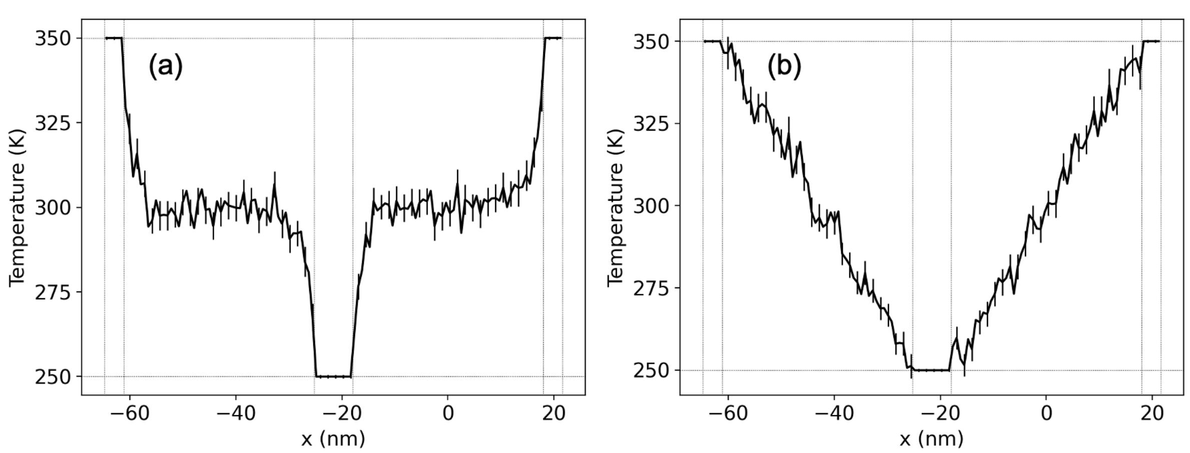

Figure S9: Temperature profile averaged over 1 ps using different relaxation procedures. (a) 50 ps of molecular dynamics with velocity rescaling in the thermal bath regions only. The target temperatures are 250 K and 350 K in the cold and hot baths, respectively and the temperature tolerance for rescaling was set to 1 K. (b) Relaxation using sequential molecular dynamics runs with decreasing thermostatting applied across the entire system, after which thermostatting outside of the bath regions is turned off entirely.

$\sqrt{\langle \Delta T^2 \rangle} = \sqrt{\frac{k_B}{C}}T \sim \frac{T}{\sqrt{N}}$, where C is the heat capacity (74). However, direct comparisons with properties of equilibrium ensembles should be approached with caution given the nonequilibrium nature of the system under consideration.

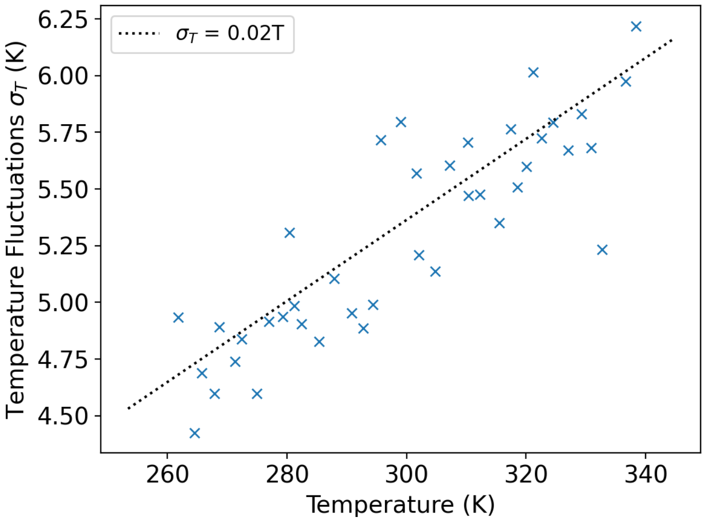

Figure S10: Root-mean-square temperature fluctuations (i.e. error bars in Fig. S8) as a function of local temperature over bins of size 1×7 unit cells, showing a weak linear dependence on the local temperature.

10 Analysis of drift velocity, $\langle v_x \rangle$

The instantaneous drift velocity at each time step and over each trajectory is calculated from the difference in centre-of-charge between time increments of 2 fs i.e. $v^t = (\langle x \rangle^{t+\delta t} - \langle x \rangle^t)/\delta t$, with $\delta t = 2$ fs. We use bins of length 4 unit cells (2.88 nm) along x (parallel to the a-crystallographic direction) and allocate each velocity to the bin which includes the initial centre-of-charge position, $\langle x \rangle^t$. The temperature gradient is 2.78 K/nm, therefore the temperature difference between the centre of adjacent bins is 8 K. The Seebeck coefficient is determined from the average velocity in the central bin (i.e. the bin centred at the position where $T = 300$ K).

Due to fluctuations in local temperature ($\approx$ 5 K, Figure S10), a temperature difference between two points in space is only well-resolved for distances larger than $\approx$ 1 nm. Therefore when velocities are very small (i.e. the change in centre-of-charge after a time increment is significantly smaller than 1 nm), there should be little contribution to the Seebeck coefficient since locally the temperature is indistinguishable on this length scale. For large velocities, the temperature profile is clearly resolved and there is a significant contribution to the Seebeck coefficient. Large velocities generally occur after a successful hop to a new active state at some distance from the old active state. The charge carrier wavefunction spatially follows the active state on average, due to the decoherence correction which damps the non-active-surface adiabatic coefficients (see main text *Methods*). As explained in the main text, the temperature gradient induces a symmetry breaking which causes the probability of surface hopping to states on the cold side to be larger than the probability of surface hopping to states on the hot side. Figure S11(a) shows a histogram of the centre-of-charge velocities, only counting velocities where the centre-of-charge lies within the central 4 unit cells along x for constant temperature simulations at $T = 300$ K and simulations under a temperature gradient, including all time steps and all trajectories.

Visually, the distributions of drift velocities for simulations at constant temperature and under a temperature gradient appear very similar. However, the mean velocity for the con-

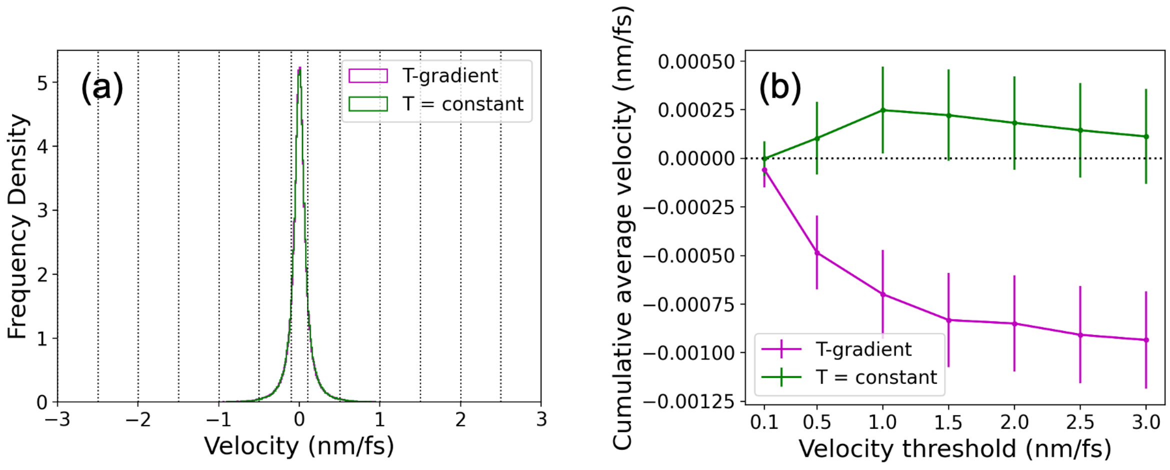

Figure S11: Analysis of the velocity distribution of the charge carrier wavefunction when its centre-of-charge is within the central 4 unit cells of the simulation box along the a crystallographic direction. (a) Histogram of velocities in the central 4 unit cells for simulations under the temperature gradient and at constant T = 300 K. (b) Cumulative average velocity, taking into account only velocities whose absolute value is smaller than a threshold.

stant temperature case is 0.10 ± 0.24 nm/ps, while the mean velocity for simulations under a temperature gradient is -0.89 ± 0.25 nm/ps. Figure S11(b) shows the cumulative drift velocity obtained by averaging over signed instantaneous drift velocities with absolute value smaller than some threshold. In the case of constant temperature simulations (green), this remains close to 0, within the margin of statistical error. For simulations under a temperature gradient, the drift velocity becomes more negative as the threshold is increased, corresponding to the net motion from hot to cold, and it is well converged when all instantaneous velocities with absolute value smaller than ≈ 1.5 nm/fs are included. Velocities larger than this are extremely rare and therefore do not affect the average significantly. Notice that the average drift velocity (i.e. the cumulative drift velocity after all velocities are included) is 2-3 orders of magnitude smaller than typical instantaneous drift velocities as positive and negative instantaneous drift velocities cancel to a large extent resulting in only relatively small net drift velocity, $\langle v_x \rangle$.

11 Analysis of thermoelectric motion

Figures S12 and S13 show distance resolved properties of the valence band states ψ_k for simulations with a temperature gradient and at constant $T = 300$ K, respectively. The Boltzmann average, indicated by $\langle ... \rangle^{\mathrm{B}}$, is defined in section *Methods* of the main text, Eq. 16. N^{conf} refers to the total number of configurations (i.e. Hamiltonians) selected for the analysis, according to the criteria that a successful hop occurs with active state located in the central 10 unit cells of the simulation box along x. Panels S12(b) and S12(d) show the same information as panels 4(b) and 4(d) of the main text, while panels S13(b) and S13(d) show the same information as panels 4(c) and 4(e) of the main text.

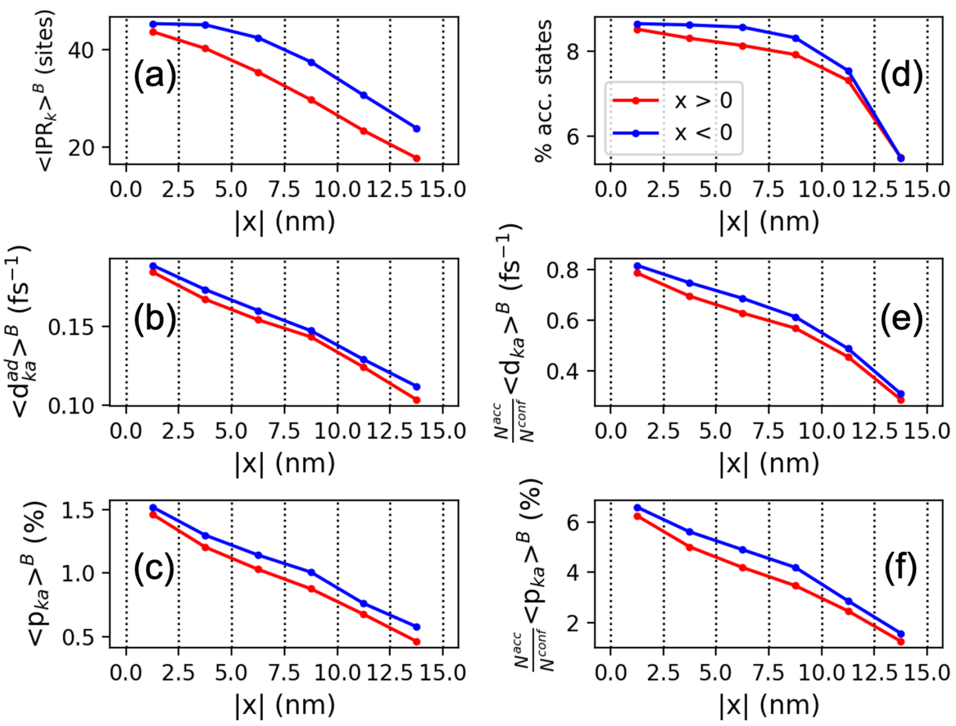

Figure S12: Position-resolved properties of valence band states ψ_k when a temperature gradient is present. Properties for states located towards the cold side and hot side, relative to the location of the current active state, are represented in red and blue, respectively. (a) Boltzmann averaged IPR, (b) Boltzmann averaged NACE, (c) Boltzmann averaged hopping probability, (d) percentage of thermally accessible states, (e) sum of Boltzmann weighted NACEs within a given position bin, (f) sum of Boltzmann weighted hopping probabilities within a given position bin.

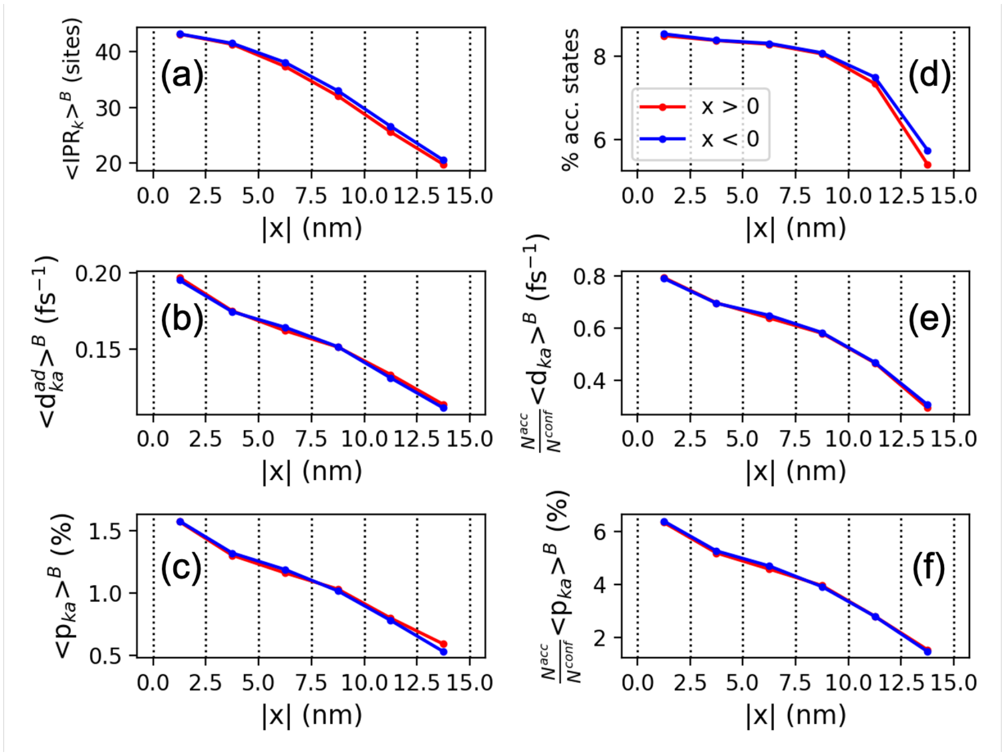

Figure S13: Position-resolved properties of valence band states ψ_k for simulations at constant $T = 300$ K. Properties for states located towards the cold side and hot side, relative to the location of the current active state, are represented in red and blue, respectively. (a) Boltzmann averaged IPR, (b) Boltzmann averaged NACE, (c) Boltzmann averaged hopping probability, (d) percentage of thermally accessible states, (e) sum of Boltzmann weighted NACEs within a given position bin, (f) sum of Boltzmann weighted hopping probabilities within a given position bin.

It is evident that in the case of constant temperature simulations (Figure S13), there is no difference between states with $\Delta\text{COC}_{ka} < 0$ compared to states with $\Delta\text{COC}_{ka} > 0$ (blue vs red lines). When a temperature gradient is applied (Figure S12), an asymmetry arises causing a clear splitting between quantities corresponding to states on the cold side compared to the hot side. $\langle d_{ka}^{\text{ad}} \rangle^{\text{B}}$ is larger for states towards the cold side ($\Delta\text{COC}_{ka} < 0$), which results in a larger Boltzmann averaged surface hopping probability, $\langle p_{ka} \rangle^{\text{B}}$, for states towards the cold side. Additionally, there is a greater number of thermally accessible states towards the cold side. These two effects result in a larger overall probability of surface hopping to any state at distance $\Delta\text{COC}_{ka} \in x$, determined by the product $\frac{N^{\text{acc}}}{N^{\text{conf}}} \times \langle p_{ka} \rangle^{\text{B}}$.

To assess the factors contributing to the higher average NACE for states on the cold

side compared to those on the hot side, we present 2D histograms in Figure S14 correlating $|d_{ka}^{\mathrm{ad}}|$ with either the inverse energy difference between states, $1/|\Delta E_{ka}|$ (panels a-d), or the product of the IPR of the active state a and state k, $\mathrm{IPR}_a \mathrm{IPR}_k$ (panels (e-h)). The NACE varies by orders of magnitude, therefore the natural logarithm of each quantity is taken. All states located in a given distance bin (towards hot and cold) relative to the position of the active state are included in the same plot, using the same binning procedure described in Figure 5 of the main text.

The non-adiabatic coupling element between adiabatic states k and l, $d_{kl}^{\mathrm{ad}} = \left\langle \psi_k \middle| \dot{\psi}_l \right\rangle$ is related to the non-adiabatic coupling vector, $\mathbf{D}_{kl}^{\mathrm{ad}}$, through the chain rule, $d_{kl}^{\mathrm{ad}} = \mathbf{D}_{kl} \cdot \frac{\partial \mathbf{R}}{\partial t}$ where(54)

$$\mathbf{D}_{kl}^{\mathrm{ad}} = \langle \psi_k | \, \partial_{\mathbf{R}} \, | \psi_l \rangle \tag{S8}$$

$$= \frac{\langle \psi_k | \, \partial_{\mathbf{R}} H \, | \psi_l \rangle}{\Delta E_{lm}}. \tag{S9}$$

Here, $\partial_{\mathbf{R}}$ denotes the derivative with respect to coordinates, H is the electronic Hamiltonian and $\Delta E_{lm} = E_l - E_m$ is the energy difference between states l and m. Equation S9 shows that the non-adiabatic coupling becomes large when states become close in energy, evident in Figure S14(a-d), where scatter is due to variation in the numerator.

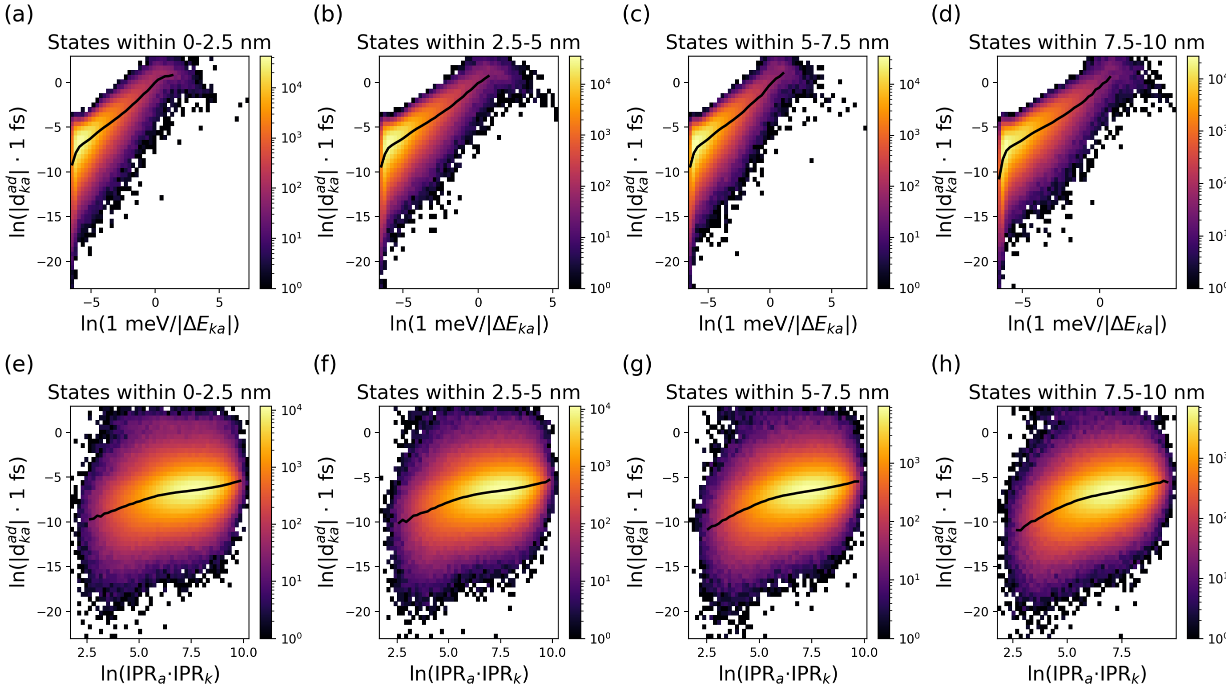

Figure S14: 2D histograms plotting the natural logarithm of the unsigned NACE, $\ln\left(|d_{ka}^{\mathrm{ad}}|\right)$, against $\ln(1/|\Delta E_{ka}|)$ (a-d), and $\ln(\mathrm{IPR}_a\mathrm{IPR}_k)$ (e-h) including states centred at different distance bins from the position of the active state (same binning procedure as used in Fig. 5 of the main text). The color scale indicates the number of states in the relevant 2D bin. Black lines in bold indicate the average value, $\langle\ln\left(|d_{ka}^{\mathrm{ad}}|\right)\rangle$. There is a clear correlation between $\ln\left(|d_{ka}^{\mathrm{ad}}|\right)$ and $\ln(1/\Delta E_{ka})$, expected due to the explicit dependence of the NACE on $1/\Delta E_{ka}$, equation S9. This results in the asymmetry in average NACE to states located on the cold side compared to the hot side (Fig. 5(b) of the main text) due to the increased likelihood of encountering small ΔE values as the density of accessible states increases from hot to cold along the temperature gradient. The correlation between the NACE and the product $\mathrm{IPR}_a\mathrm{IPR}_k$ is smaller (albeit non-zero), indicated that state delocalization is a less significant factor determining the magnitude of the NACE.

12 Convergence of the kinetic contribution to Seebeck coefficient, α_v

Simulations were carried out with a temperature gradient and no external field (denoted T-gradient), at constant temperature $T = 300$ K (i.e. the control simulations, denoted $T =$ constant) and with a temperature gradient and an external field (denoted T-gradient + E-field). In each case, we ran a total of 2000 FOB-SH trajectories. For T-gradient and $T =$ constant simulations, trajectories were of length 5 ps (i.e. a total of 10 ns of dynamics), whereas for the T-gradient + E-field, trajectories were run to 3 ps (i.e. a total of 6 ns of dynamics). In each case, the wavefunction was initialised from 5 different positions spread uniformly along the x-direction of the active region in FOB-SH simulations, i.e., 400 trajectories per initial condition. The kinetic contribution to the Seebeck coefficient, α_v, is calculated from the average drift velocity of the charge carrier in the central position bin, $\alpha_v = -\frac{\langle v_x \rangle}{\mu \partial_x T}$ (first term on the RHS of Eq. 2 in the main text). Figure S15 shows the convergence of $\langle v_x \rangle$ for the different simulations with (a) number of trajectories (including their full length) and (b) trajectory length using all 2000 trajectories. Error bars represent the standard error of the velocity distribution in the central bin, $\sigma_v/\sqrt{N}$, where σ_v is the standard deviation of velocities observed in the central bin and N is the number of data points. For the T-gradient simulations (magenta), using just 50 trajectories per initial condition (i.e. 250 trajectories overall) already yields a mean value close to the final result, however the relatively small amount of data leads to a large error in the mean. Using trajectories of length shorter than 3 ps leads to an overly negative value for $\langle v_x \rangle$ (i.e. an overestimation of α_v). This is because trajectories which start from the hot side reach the central bin (where drift velocities are used in the average) faster than trajectories starting from the cold end, leading to a small amount of bias. α_v is well converged if trajectories have length 3 ps or longer. For the $T =$ constant simulations (green) and T-gradient + E-field simulations (blue), the average velocity in the central bin is approximately converged using around 100 trajectories per initial condition

(i.e. 500 trajectories overall). As described in the main text, the electric field is chosen to compensate for the net drift velocity observed in the T-gradient simulations such that open-circuit conditions are achieved. This is clearly illustrated in Figure S15; when the electric field is included, the drift velocity in the central bin becomes close to zero (approximately matching the situation observed for T = constant simulations).

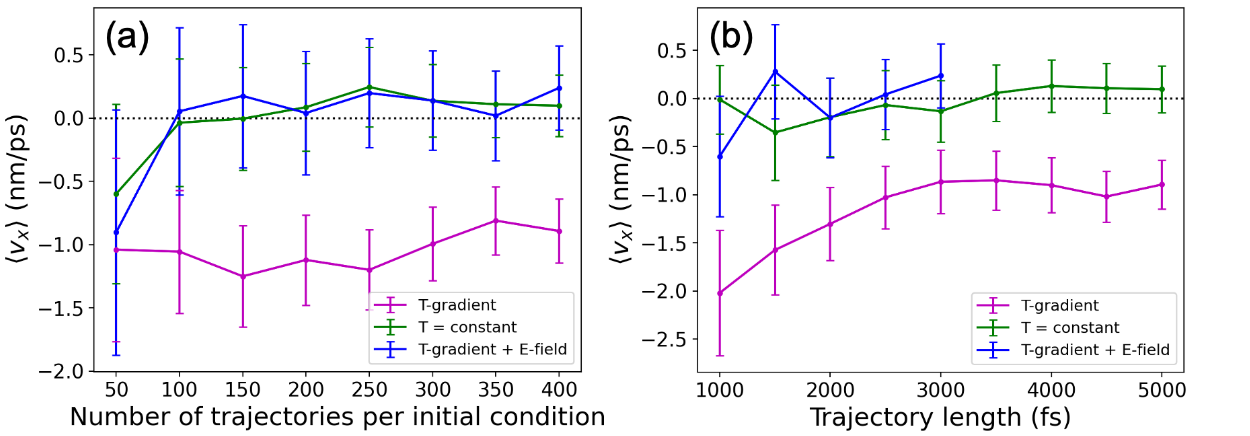

Figure S15: Convergence of the average drift velocity in the central position bin, $\langle v_x \rangle$, for simulations employing a temperature gradient with no external E-field (magenta), constant temperature $T = 300$ K (green) and a temperature gradient with an external E-field chosen to cancel the kinetic contribution to the Seebeck coefficient (blue), i.e. same colour scheme as Figure 3 of the main text. (a) Convergence with number of trajectories per initial position of the hole wavefunction. (b) Convergence with trajectory length using all 2000 trajectories. For T-gradient and T = constant simulations, trajectories were run to 5 ps, whereas for T-gradient + E-field simulations, trajectories were run to 3 ps.

13 Chemical Potential Contribution to Seebeck coefficient, α_c

As described in the main text *Methods*, the chemical potential contribution to the Seebeck coefficient (second term on the RHS of equation 2 of the main text) is given by

$$\alpha_c = -\frac{1}{q}\frac{\partial_x \mu_c}{\partial_x T} = -\frac{1}{q}\frac{\partial \mu_c}{\partial T}, \tag{S10}$$

where we have used the chain rule in the second equation of equation S10. The chemical potential at temperature T and reference carrier concentration n^{ref} is given by the change in free energy upon insertion of a charge carrier into the band (Eq. 18 of the main text):

$$\mu_c^{\mathrm{ref}}(T, n^{\mathrm{ref}}) = F_{\mathrm{hole}}(T, n^{\mathrm{ref}}) - F_{\mathrm{neutral}}(T, n^{\mathrm{ref}}) \tag{S9}$$

$$= -k_B T[\ln(Z_{\mathrm{hole}}) - \ln(Z_{\mathrm{neutral}})] \tag{S10}$$

$$= -k_B T \ln \frac{\int d\mathbf{R} \sum_i^{\mathrm{vb}} e^{+\beta E_i(\mathbf{R})}}{\int d\mathbf{R} e^{-\beta E_{\mathrm{neutral}}(\mathbf{R})}} \tag{S11}$$

$$= -k_B T \ln \frac{\int d\mathbf{R} \sum_i^{\mathrm{vb}} e^{+\beta[E_i(\mathbf{R})+E_{\mathrm{neutral}}(\mathbf{R})]} e^{-\beta E_{\mathrm{neutral}}(\mathbf{R})}}{\int d\mathbf{R} e^{-\beta E_{\mathrm{neutral}}(\mathbf{R})}} \tag{S12}$$

$$= -k_B T \ln \left\langle \sum_i^{\mathrm{vb}} e^{+\beta[E_i(\mathbf{R})+E_{\mathrm{neutral}}(\mathbf{R})]} \right\rangle_{E_{\mathrm{neutral}}(\mathbf{R})}^{n^{\mathrm{ref}}}, \tag{S13}$$

where F denotes free energy, Z is the partition function, $\mathbf{R}$ denotes nuclear coordinates, $E_i(\mathbf{R})$ is the ith eigenstate of the FOB-SH Hamiltonian describing an excess hole at nuclear geometry $\mathbf{R}$ and E_{neutral} is the energy of the neutral system at nuclear geometry $\mathbf{R}$. Note the + sign in the Boltzmann weight over valence band (vb) states, indicating that increasing excitation corresponds to decreased energy. The brackets denote taking the thermal average, which can be obtained by sampling nuclear configurations from a molecular dynamics trajectory of the system in the charge-neutral state at carrier density n^{ref}. The chemical potential at temperature T and general carrier density n is given by equation 19 of the main

text, reproduced here for clarity:

$$\mu_c(T,n) = \mu_c^{\text{ref}}(T,n^{\text{ref}}) + k_B T \ln \frac{n}{n^{\text{ref}}}. \tag{S14}$$

Taking the derivative with respect to temperature:

$$\frac{\partial \mu_c(T,n)}{\partial T} = \frac{\partial \mu_c^{\text{ref}}(T,n^{\text{ref}})}{\partial T} + k_B \ln \frac{n}{n^{\text{ref}}}, \tag{S15}$$

from which α_c may be calculated using equation S10:

$$\alpha_c(T,n) = -\frac{1}{q}\frac{\partial \mu_c}{\partial T} = -\frac{1}{q}\left[\frac{\partial \mu_c^{\text{ref}}}{\partial T} + k_B \ln \frac{n}{n^{\text{ref}}}\right]. \tag{S16}$$

To obtain the first term on the RHS of equation S16, $\alpha_c^{\text{ref}} = -\frac{1}{q}\frac{\partial \mu_c^{\text{ref}}(T,n^{\text{ref}})}{\partial T}$ at 300 K, the chemical potential μ_c^{ref} was calculated for a given reference concentration n^{ref} (see below) at temperatures $T = 275, 300, 325$ K according to equation S13 using MD simulation on the neutral rubrene crystal with a simulation box of $50 \times 7 \times 1$ unit cells (the same size used for FOB-SH simulations with a temperature gradient). The temperature derivative was then obtained from the best slope fit. In order to verify that the chemical potential (Eq. S14) and Seebeck coefficient (Eq. S16) are independent of the chosen value for n^{ref}, α_c^{ref} was calculated at 6 different values of $n^{\text{ref}} = 1/A$, where A is the area of the active region parallel to the a-b crystallographic plane. The active region is the part of the supercell for which the valence band electronic Hamiltonian (Eq. 4 main text) is constructed. The values of n^{ref} used, along with the corresponding active region sizes and results for μ_c^{ref} and α_c^{ref} are listed in Table S8. The uncertainty in α_c^{ref} represents one standard deviation, calculated from the square root of the relevant diagonal value of the covariance matrix for the fit parameters.

Figure S16 plots the calculated values of $\alpha_c(T=300\text{ K}, n)$, equation S16, for the different definitions of n^{ref} listed in Table S8, at the concentration n^{ref}, i.e., $\alpha_c(T=300\text{K}, n=n^{\text{ref}}) = -\frac{1}{q}\left[\frac{\partial \mu_c^{\text{ref}}}{\partial T} + k_B \ln \frac{n^{\text{ref}}}{n^{\text{ref}}}\right] = -\frac{1}{q}\left[\frac{\partial \mu_c^{\text{ref}}}{\partial T}\right] = \alpha_c^{\text{ref}}$ (black crosses). The best linear fit of α_c^{ref} to $\ln(n)$ is

indicated with the dotted back line. The magenta dashed line shows the carrier concentration dependence of α_c from equation S16 when a value of $n^{\mathrm{ref}} = 2.74 \times 10^{15}\,\mathrm{m}^{-2}$ is chosen (i.e. a simulation box of $50 \times 7 \times 1$ unit cells). The explicitly calculated data points (black crosses) coincide very well with the analytic expression equation S16 (within a single error bar). The slope of the best fit line (dotted black) is $\partial\alpha_c/\partial \ln(n) = -82.9\ \mu$V/K, in very close agreement with the expected slope from equation S16, $-\frac{k_B}{q} = -86.2\ \mu$V/K, implying that the thermodynamic theory and simulation provide a fully consistent description of the concentration dependence of α_c which does not depend on the chosen reference density n^{ref}.

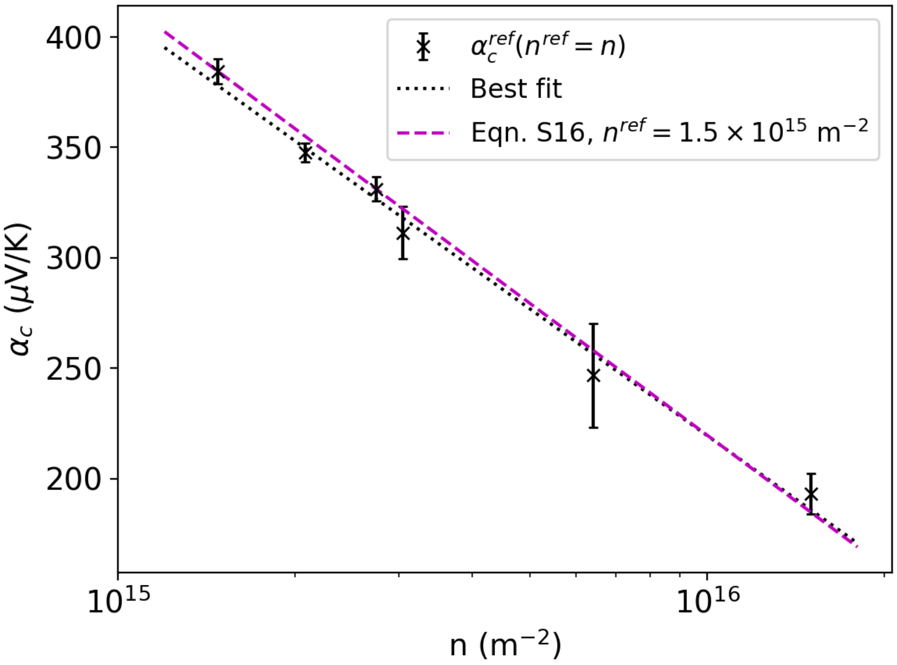

Figure S16: Chemical potential contribution to the Seebeck coefficient, α_c, as a function of carrier concentration, n. α_c^{ref} calculated for the concentrations shown on the x-axis, i.e. $n^{\mathrm{ref}} = n$, are shown as black crosses (values taken from Table S8), with the best linear fit of α_c^{ref} to $\ln(n)$ indicated with the dotted black line. The analytic dependence of α_c on concentration (Eq. S16) is shown in dashed magenta, where a single value for α_c^{ref} at $n^{\mathrm{ref}} = 2.74 \times 10^{15}\,\mathrm{m}^{-2}$ was used. See text for details. The explicitly calculated data points (black crosses) align extremely well with the analytic expression given by equation S16 across the entire concentration range. The slopes $\partial\alpha_c/\partial \ln(n)$ for the best fit line and equation S16 are $-82.9\ \mu$V/K and $-\frac{k_B}{q} = -86.2\ \mu$V/K, respectively, validating the concentration dependence in equation S16 and showing that $\alpha_c(T, n)$ does not depend on the chosen reference density n^{ref}.

Table S8: Calculated values for the chemical potential μ_c^{ref} at different values of n^{ref} corresponding to different active region sizes for temperatures $T = 275, 300, 325$ K and the resulting value for α_c^{ref}.

n^{ref} (m^{-2})	Active Region (unit cells)	$\mu_c^{\mathrm{ref}}\|_{275\mathrm{K}}$ (meV)	$\mu_c^{\mathrm{ref}}\|_{300\mathrm{K}}$ (meV)	$\mu_c^{\mathrm{ref}}\|_{325\mathrm{K}}$ (meV)	α_c^{ref} (μV/K)
1.5×10^{15}	50×13	-315.1	-324.5	-334.3	384.5 ± 5.5
2.1×10^{15}	42×11	-306.5	-315.1	-323.9	347.7 ± 4.4
2.7×10^{15}	50×7	-300.0	-308.0	-316.5	331.3 ± 5.6
3.0×10^{15}	35×9	-297.3	-304.6	-312.9	311.3 ± 11.9
6.4×10^{15}	25×6	-279.1	-284.2	-291.4	246.8 ± 23.6
1.5×10^{16}	16×4	-255.7	-260.1	-265.3	193.2 ± 9.1

14 Experimental Determination of Mobility and Seebeck Coefficient

A schematic of the electrode pattern used, and an image of the actual device are given in Figure S17.

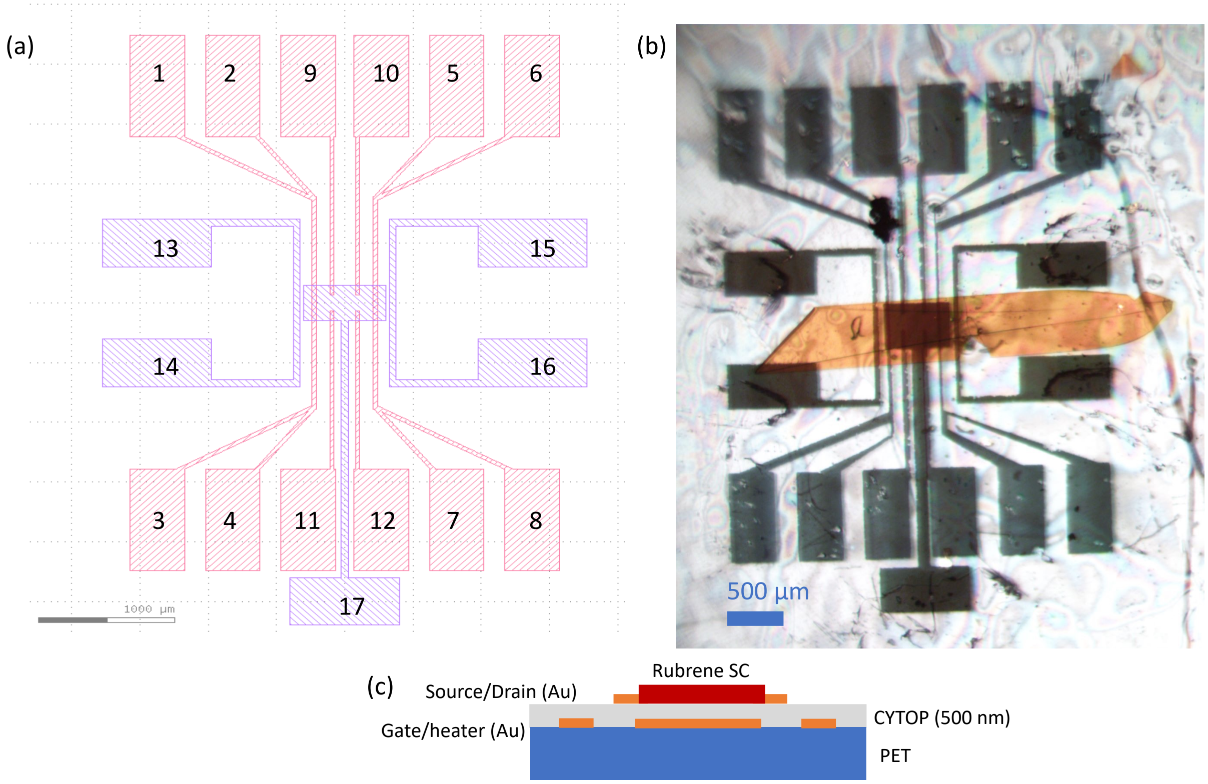

Figure S17: Schematics and image of the experimental device used to measure the Seebeck coefficient. (a) Shadow mask pattern used for measuring mobility and Seebeck coefficient in rubrene single crystals. The electrodes in blue are evaporated directly on the PET, while those in red are evaporated on the CYTOP dielectric, as shown in panel (c). Contacts 1–4 connect to the source/hot thermometer; 5–8 to the drain/cold thermometer; 9–12 are used as the voltage probes for the 4-point probe mobility measurements; 13–16 allow either heater wire to be used; 17 is the gate electrode. (b) Micrograph of the finished device with rubrene single crystal. (c) Side view showing the layers of transistor architecture.

The mobility of the rubrene single crystals is determined via a 4-point probe method in FET geometry. The crystals show an increasing mobility with decreasing temperature, indicative of band-like transport, as well as a turn on voltage close to 0 V suggesting a very

low deep trap density.

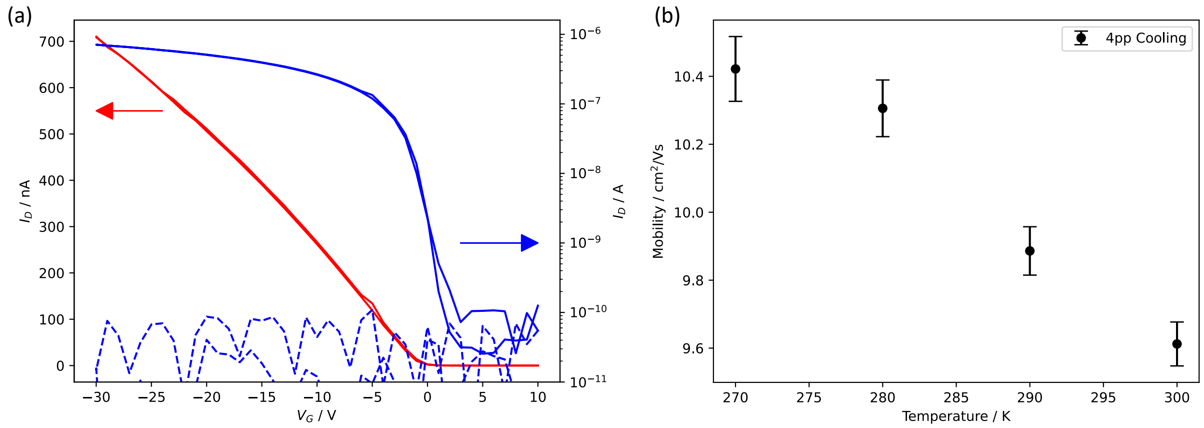

Figure S18: Transfer properties of the rubrene single crystal (a) The measured transfer curve in both linear (red) and log (blue) current scale. Solid lines show the source-drain current while dashed line shows the source-gate current. Here $V_{SD} = -1$ V. (b) The experimental mobility vs. temperature curve measured *via* 4-point probe method.

As we cool down the temperature from 300 K to 270 K, the threshold voltage shifts from near 0 V to -1 V indicating the existence of some trap states.

In order to calculate the Seebeck coefficient of a material, the generated voltage across a known thermal gradient is measured. As described in previous works (65), in this study, the thermal gradient is created via resistive heating by on-device heater electrodes, with applied heater power W,

$$\alpha = \frac{\Delta V}{\Delta T} = \frac{\frac{\Delta V}{\Delta W}}{\frac{\Delta T}{\Delta W}}. \tag{S11}$$

To find the absolute temperature difference between the two ends, the resistance of the source and drain electrodes are measured by a 4-point probe method. We apply a current across one pair of electrodes (e.g. 1 and 3) and measure the voltage across the other pair (2 and 4), which negates any contact resistance from the probes or resistance from cabling. Because the source and drain are gold – which has a linear resistivity vs. temperature curve – the applied temperature at the hot and cold ends can be found by measuring the resistance (R) as a function of applied heater power. Finally, each thermometer needs calibrating by

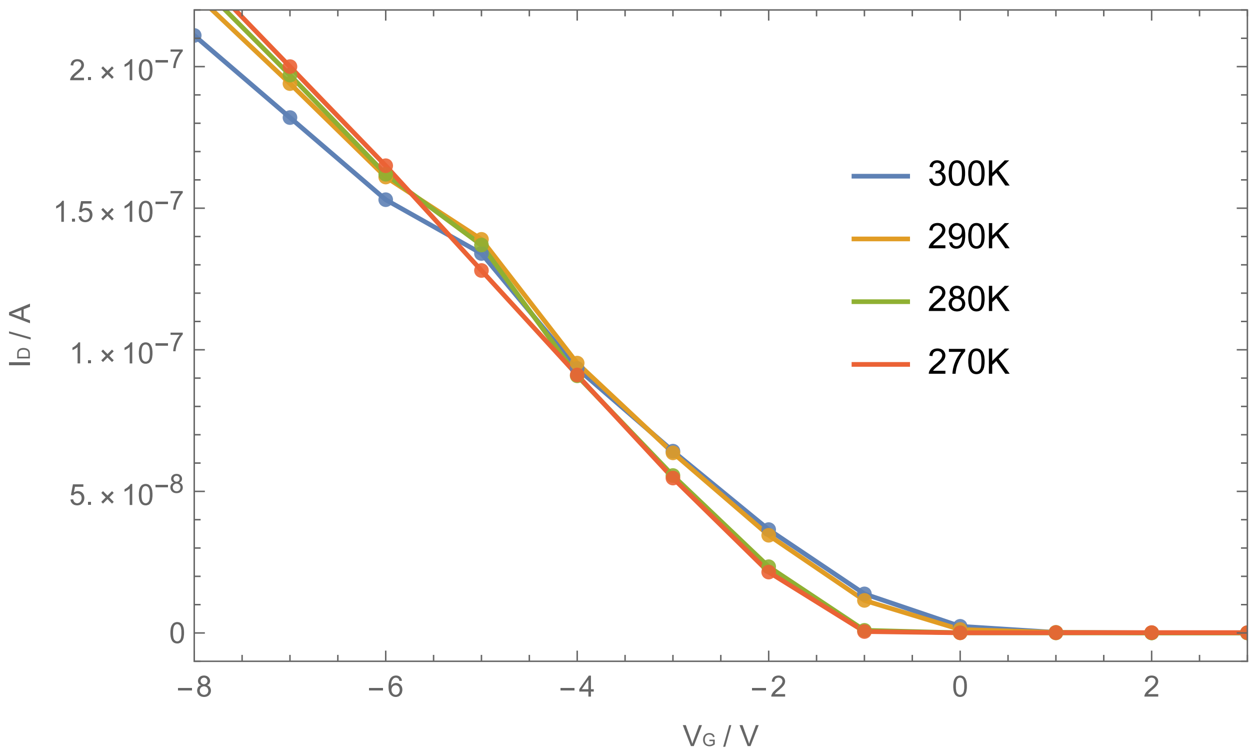

Figure S19: Linear regime transfer characteristics around the turn on voltage at temperatures from 300 K to 270 K.

measuring its resistance at varying cryostat base temperatures T.

In this way:

$$\frac{\Delta T}{\Delta W} = \frac{dT}{dW}_{hot} - \frac{dT}{dW}_{cold}, \tag{S12}$$

and

$$\frac{dT}{dW}_{hot,cold} = \frac{\frac{dR}{dW}_{hot,cold}}{\frac{dR}{dT}_{hot,cold}}. \tag{S13}$$

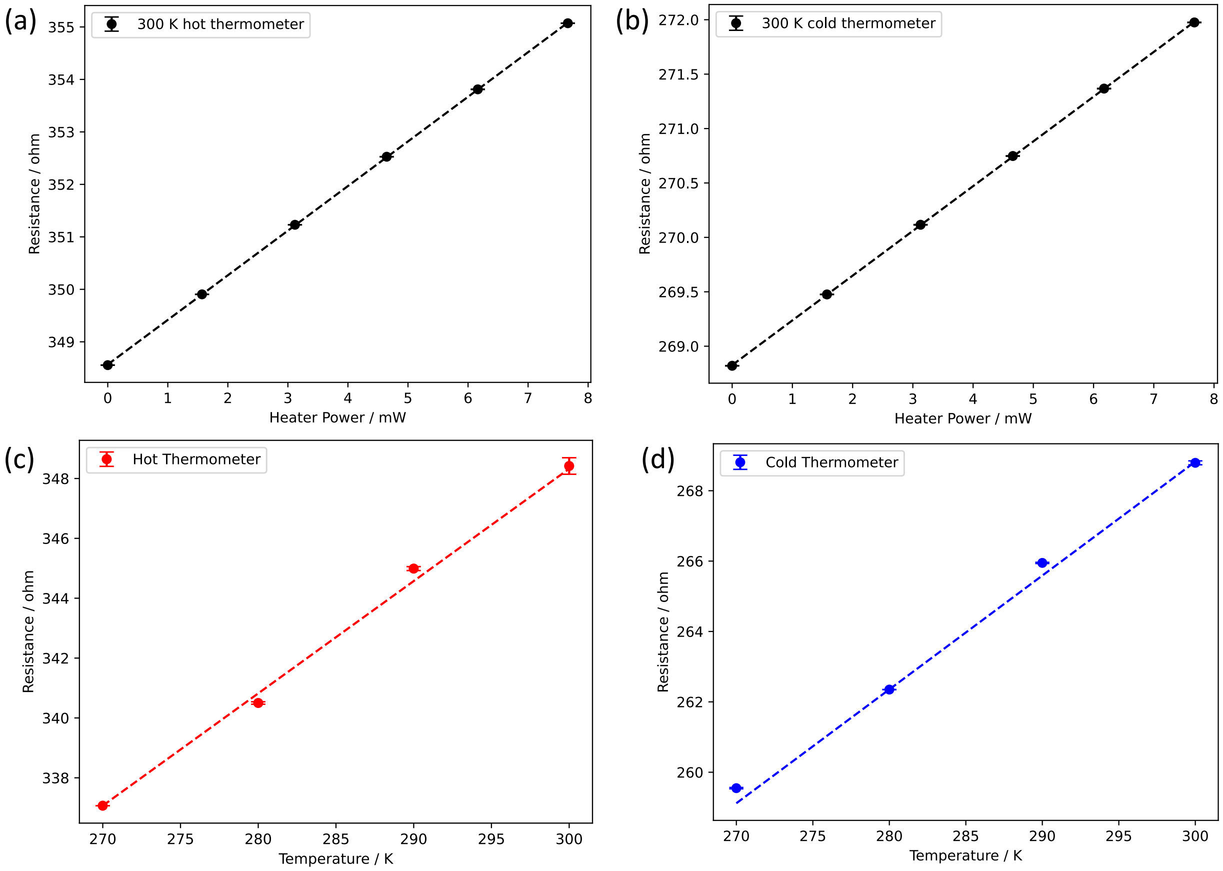

Figure S20: Hot and cold sensor calibrations for determining the temperature difference across the device. (a) and (b) show the R vs. W curves at 300 K, while (c) and (d) show the sensor wire resistances as a function of cryostat temperature.

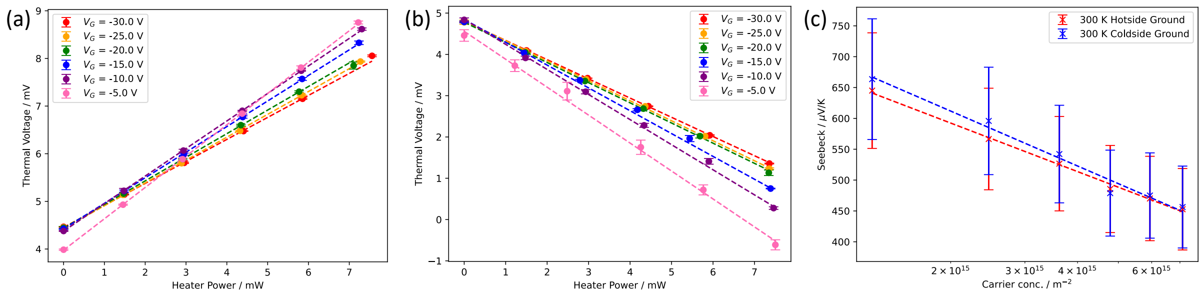

Figure S21: Thermal voltage vs. heater power for the rubrene crystal with both the hot side grounded (a) and the cold side grounded (b). As expected for devices with no leakage both give the same magnitude of gradient except inverted, and so the extracted Seebeck coefficient is the same (c). Note that the voltage offset in (a) and (b) is due to the pre-amplifier used with the measuring SMU.

REFERENCES AND NOTES

1. N. Lu, L. Li, M. Liu, A review of carrier thermoelectric-transport theory in organic semiconductors. *Phys. Chem. Chem. Phys.* **18**, 19503–19525 (2016).

2. F. Zhang, C.-a. Di, Exploring thermoelectric materials from high mobility organic semiconductors. *Chem. Mater.* **32**, 2688–2702 (2020).

3. D. Venkateshvaran, M. Nikolka, A. Sadhanala, V. Lemaur, M. Zelazny, M. Kepa, M. Hurhangee, A. J. Kronemeijer, V. Pecunia, I. Nasrallah, I. Romanov, K. Broch, I. McCulloch, D. Emin, Y. Olivier, J. Cornil, D. Beljonne, H. Sirringhaus, Approaching disorder-free transport in high-mobility conjugated polymers. *Nature* **515**, 384–388 (2014).

4. G.-H. Kim, L. Shao, K. Zhang, K. P. Pipe, Engineered doping of organic semiconductors for enhanced thermoelectric efficiency. *Nat. Mater.* **12**, 719–723 (2013).

5. A. Gemma, F. Tabatabaei, U. Drechsler, A. Zulji, H. Dekkiche, N. Mosso, T. Niehaus, M. R. Bryce, S. Merabia, B. Gotsmann, Full thermoelectric characterization of a single molecule. *Nat. Commun.* **14**, 3868 (2023).

6. S. Masoumi, S. O'Shaughnessy, A. Pakdel, Organic-based flexible thermoelectric generators: From materials to devices. *Nano Energy* **92**, 106774 (2022).

7. A. Zevalkink, D. M. Smiadak, J. L. Blackburn, A. J. Ferguson, M. L. Chabinyc, O. Delaire, J. Wang, K. Kovnir, J. Martin, L. T. Schelhas, T. D. Sparks, S. D. Kang, M. T. Dylla, G. J. Snyder, B. R. Ortiz, E. S. Toberer, A practical field guide to thermoelectrics: Fundamentals, synthesis, and characterization. *Appl. Phys. Rev.* **5**, 021303 (2018).

8. M. S. Lundstrom, C. Jeong, *Near-Equilibrium Transport: Fundamentals and Applications* (World Scientific Publishing Company, 2012), vol. 2.

9. C. Jeong, R. Kim, M. Luisier, S. Datta, M. Lundstrom, On Landauer versus Boltzmann and full band versus effective mass evaluation of thermoelectric transport coefficients. *J. Appl. Phys.* **107**, 023707 (2010).

10. D. Wang, W. Shi, J. Chen, J. Xi, Z. Shuai, Modeling thermoelectric transport in organic materials. *Phys. Chem. Chem. Phys.* **14**, 16505–16520 (2012).

11. L. Friedman, Transport properties of organic semiconductors. *Phys. Rev.* **133**, A1668–A1679 (1964).

12. T. Mori, H. Inokuchi, Thermoelectric power of organic superconductors—Calculation on the basis of the tight-binding theory. *J. Phys. Soc. Jpn.* **57**, 3674–3677 (1988).

13. I. G. Austin, N. F. Mott, Polarons in crystalline and non-crystalline materials. *Adv. Phys.* **18**, 41–102 (1969).

14. P. M. Chaikin, G. Beni, Thermopower in the correlated hopping regime. *Phys. Rev. B* **13**, 647–651 (1976).

15. D. Emin, Thermoelectric power due to electronic hopping motion. *Phys. Rev. Lett.* **35**, 882–885 (1975).

16. D. Emin, Enhanced Seebeck coefficient from carrier-induced vibrational softening. *Phys. Rev. B* **59**, 6205–6210 (1999).

17. D. Emin, "Seebeck effect" in *Wiley Encyclopedia of Electrical and Electronics Engineering* (Wiley, 2014), pp. 1–18.

18. A. Troisi, G. Orlandi, Charge-transport regime of crystalline organic semiconductors: Diffusion limited by thermal off-diagonal electronic disorder. *Phys. Rev. Lett.* **96**, 086601 (2006).

19. S. Fratini, D. Mayou, S. Ciuchi, The transient localization scenario for charge transport in crystalline organic materials. *Adv. Funct. Mater.* **26**, 2292–2315 (2016).

20. S. Fratini, S. Ciuchi, D. Mayou, G. T. de Laissardière, A. Troisi, A map of high mobility molecular semiconductors. *Nat. Mater.* **16**, 998–1002 (2017).

21. S. Fratini, M. Nikolka, A. Salleo, G. Schweicher, H. Sirringhaus, Charge transport in high-mobility conjugated polymers and molecular semiconductors. *Nat. Mater.* **19**, 491–502 (2020).

22. S. Few, J. M. Frost, J. Nelson, Models of charge pair generation in organic solar cells. *Phys. Chem. Chem. Phys.* **17**, 2311–2325 (2015).

23. L. Wang, D. Beljonne, Flexible surface hopping approach to model the crossover from hopping to band-like transport in organic crystals. *J. Phys. Chem. Lett.* **4**, 1888–1894 (2013).

24. L. Wang, O. V. Prezhdo, D. Beljonne, Mixed quantum-classical dynamics for charge transport in organics. *Phys. Chem. Chem. Phys.* **17**, 12395–12406 (2015).

25. S. Giannini, L. di Virgilio, M. Bardini, J. Hausch, J. J. Geuchies, W. Zheng, M. Volpi, J. Elsner, K. Broch, Y. H. Geerts, F. Schreiber, G. Schweicher, H. I. Wang, J. Blumberger, M. Bonn, D. Beljonne, Transiently delocalized states enhance hole mobility in organic molecular semiconductors. *Nat. Mater.* **22**, 1361–1369 (2023).

26. A. Heck, J. J. Kranz, T. Kubař, M. Elstner, Multi-scale approach to non-adiabatic charge transport in high-mobility organic semiconductors. *J. Chem. Theory Comput.* **11**, 5068–5082 (2015).

27. W. Xie, D. Holub, T. Kubař, M. Elstner, Performance of mixed quantum-classical approaches on modeling the crossover from hopping to bandlike charge transport in organic semiconductors. *J. Chem. Theory Comput.* **16**, 2071–2084 (2020).

28. S. Roosta, F. Ghalami, M. Elstner, W. Xie, Efficient surface hopping approach for modeling charge transport in organic semiconductors. *J. Chem. Theory Comput.* **18**, 1264–1274 (2022).

29. N. B. Taylor, I. Kassal, Generalised Marcus theory for multi-molecular delocalised charge transfer. *Chem. Sci.* **9**, 2942–2951 (2018).

30. D. Balzer, T. J. A. M. Smolders, D. Blyth, S. N. Hood, I. Kassal, Delocalised kinetic Monte Carlo for simulating delocalisation-enhanced charge and exciton transport in disordered materials. *Chem. Sci.* **12**, 2276–2285 (2021).

31. J. T. Willson, W. Liu, D. Balzer, I. Kassal, Jumping kinetic Monte Carlo: Fast and accurate simulations of partially delocalized charge transport in organic semiconductors. *J. Phys. Chem. Lett.* **14**, 3757–3764 (2023).

32. A. J. Sneyd, T. Fukui, D. Paleček, S. Prodhan, I. Wagner, Y. Zhang, J. Sung, S. M. Collins, T. J. A. Slater, Z. Andaji-Garmaroudi, L. R. MacFarlane, J. D. Garcia-Hernandez, L. Wang, G. R. Whittell, J. M. Hodgkiss, K. Chen, D. Beljonne, I. Manners, R. H. Friend, A. Rao, Efficient energy transport in an organic semiconductor mediated by transient exciton delocalization. *Sci. Adv.* **7**, eabh4232 (2021).

33. A. J. Sneyd, D. Beljonne, A. Rao, A new frontier in exciton transport: Transient delocalization. *J. Phys. Chem. Lett.* **13**, 6820–6830 (2022).

34. S. Giannini, A. Carof, J. Blumberger, Crossover from hopping to band-like charge transport in an organic semiconductor model: Atomistic nonadiabatic molecular dynamics simulation. *J. Phys. Chem. Lett.* **9**, 3116–3123 (2018).

35. S. Giannini, A. Carof, M. Ellis, H. Yang, O. G. Ziogos, S. Ghosh, J. Blumberger, Quantum localization and delocalization of charge carriers in organic semiconducting crystals. *Nat. Commun.* **10**, 3843 (2019).

36. S. Giannini, O. G. Ziogos, A. Carof, M. Ellis, J. Blumberger, Flickering polarons extending over ten nanometres mediate charge transport in high-mobility organic crystals. *Adv. Theory Simul.* **3**, 2000093 (2020).

37. S. Giannini, J. Blumberger, Charge transport in organic semiconductors: The perspective from nonadiabatic molecular dynamics. *Acc. Chem. Res.* **55**, 819–830 (2022).

38. J. C. Tully, Molecular dynamics with electronic transitions. *J. Chem. Phys.* **93**, 1061–1071 (1990).

39. Y. Wang, Y.-J. Hu, B. Bocklund, S.-L. Shang, B.-C. Zhou, Z.-K. Liu, L.-Q. Chen, First-principles thermodynamic theory of Seebeck coefficients. *Phys. Rev. B* **98**, 224101 (2018).

40. Y. Apertet, H. Ouerdane, C. Goupil, P. Lecoeur, A note on the electrochemical nature of the thermoelectric power. *Eur. Phys. J. Plus* **131**, 76 (2016).

41. G. Wu, X. Yu, Contributions of chemical potential to the diffusive Seebeck coefficient for bulk semiconductor materials. *Eur. Phys. J. Plus* **135**, 472 (2020).

42. J. Spencer, F. Gajdos, J. Blumberger, FOB-SH: Fragment orbital-based surface hopping for charge carrier transport in organic and biological molecules and materials. *J. Chem. Phys.* **145**, 064102 (2016).

43. A. Carof, S. Giannini, J. Blumberger, How to calculate charge mobility in molecular materials from surface hopping non-adiabatic molecular dynamics–beyond the hopping/band paradigm. *Phys. Chem. Chem. Phys.* **21**, 26368–26386 (2019).

44. S. Giannini, A. Carof, M. Ellis, O. G. Ziogos, J. Blumberger, *Multiscale Dynamics Simulations: Nano- and Nano-bio Systems in Complex Environments* (Royal Society of Chemistry, 2021), pp. 172–202.

45. F. Gajdos, S. Valner, F. Hoffmann, J. Spencer, M. Breuer, A. Kubas, M. Dupuis, J. Blumberger, Ultrafast estimation of electronic couplings for electron transfer between π-conjugated organic molecules. *J. Chem. Theory Comput.* **10**, 4653–4660 (2014).

46. O. G. Ziogos, J. Blumberger, Ultrafast estimation of electronic couplings for electron transfer between pi-conjugated organic molecules. II. *J. Chem. Phys.* **155**, 244110 (2021).

47. J. Klimeš, D. R. Bowler, A. Michaelides, Chemical accuracy for the van der Waals density functional. *J. Phys. Condens. Matter* **22**, 022201 (2010).

48. Z. Futera, J. Blumberger, Electronic couplings for charge transfer across molecule/metal and molecule/semiconductor interfaces: Performance of the projector operator-based diabatization approach. *J. Phys. Chem. C* **121**, 19677–19689 (2017).

49. O. D. Jurchescu, A. Meetsma, T. T. M. Palstra, Low-temperature structure of rubrene single crystals grown by vapor transport. *Acta Crystallogr. B* **62**, 330–334 (2006).

50. V. Podzorov, E. Menard, A. Borissov, V. Kiryukhin, J. A. Rogers, M. E. Gershenson, Intrinsic charge transport on the surface of organic semiconductors. *Phys. Rev. Lett.* **93**, 086602 (2004).

51. Z. Li, S. Xiong, C. Sievers, Y. Hu, Z. Fan, N. Wei, H. Bao, S. Chen, D. Donadio, T. Ala-Nissila, Influence of thermostatting on nonequilibrium molecular dynamics simulations of heat conduction in solids. *J. Chem. Phys.* **151**, 234105 (2019).

52. A. F. Ioffe, L. S. Stil'Bans, E. K. Iordanishvili, T. S. Stavitskaya, A. Gelbtuch, G. Vineyard, Semiconductor thermoelements and thermoelectric cooling. *Phys. Today* **12**, 42 (1959).

53. A. Carof, S. Giannini, J. Blumberger, Detailed balance, internal consistency, and energy conservation in fragment orbital-based surface hopping. *J. Chem. Phys.* **147**, 214113 (2017).

54. R. Baer, Non-adiabatic couplings by time-dependent density functional theory. *Chem. Phys. Lett.* **364**, 75–79 (2002).

55. W. Domcke, D. Yarkony, H. Koppel, *Conical Intersections: Electronic Structure, Dynamics & Spectroscopy* (World Scientific, 2004), vol. 15.

56. H. B. Callen, The application of Onsager's reciprocal relations to thermoelectric, thermomagnetic, and galvanomagnetic effects. *Phys. Rev.* **73**, 1349–1358 (1948).

57. G. D. Mahan, Density variations in thermoelectrics. *J. Appl. Phys.* **87**, 7326–7332 (2000).

58. K. P. Pernstich, B. Rössner, B. Batlogg, Field-effect-modulated Seebeck coefficient in organic semiconductors. *Nat. Mater.* **7**, 321–325 (2008).

59. A. Abutaha, P. Kumar, E. Yildirim, W. Shi, S.-W. Yang, G. Wu, K. Hippalgaonkar, Correlating charge and thermoelectric transport to paracrystallinity in conducting polymers. *Nat. Commun.* **11**, 1737 (2020).

60. Y. Lu, J.-Y. Wang, J. Pei, Achieving efficient n-doping of conjugated polymers by molecular dopants. *Acc. Chem. Res.* **54**, 2871–2883 (2021).

61. M. Ellis, H. Yang, S. Giannini, O. G. Ziogos, J. Blumberger, Impact of nanoscale morphology on charge carrier delocalization and mobility in an organic semiconductor. *Adv. Mater.* **33**, 2104852 (2021).

62. R. Hafizi, J. Elsner, J. Blumberger, Ultrafast electronic coupling estimators: Neural networks versus physics-based approaches. *J. Chem. Theory Comput.* **19**, 4232–4242 (2023).

63. J. Elsner, S. Giannini, J. Blumberger, Mechanoelectric response of single-crystal rubrene from Ab initio molecular dynamics. *J. Phys. Chem. Lett.* **12**, 5857–5863 (2021).

64. T. D. Kühne, M. Iannuzzi, M. D. Ben, V. V. Rybkin, P. Seewald, F. Stein, T. Laino, R. Z. Khaliullin, O. Schütt, F. Schiffmann, D. Golze, J. Wilhelm, S. Chulkov, M. H. Bani-Hashemian, V. Weber, U. Borštnik, M. Taillefumier, A. S. Jakobovits, A. Lazzaro, H. Pabst, T. Müller, R. Schade, M. Guidon, S. Andermatt, N. Holmberg, G. K. Schenter, A. Hehn, A. Bussy, F. Belleflamme, G. Tabacchi, A. Glöß, M. Lass, I. Bethune, C. J. Mundy, C. Plessl, M. Watkins, J. V. Vondele, M. Krack, J. Hutter, CP2K: An electronic structure and molecular dynamics software package-Quickstep: Efficient and accurate electronic structure calculations. *J. Chem. Phys.* **152**, 194103 (2020).

65. M. Statz, S. Schneider, F. J. Berger, L. Lai, W. A. Wood, M. Abdi-Jalebi, S. Leingang, H.-J. Himmel, J. Zaumseil, H. Sirringhaus, Charge and thermoelectric transport in polymer-sorted semiconducting single-walled carbon nanotube networks. *ACS Nano* **14**, 15552–15565 (2020).

66. I. N. Hulea, S. Fratini, H. Xie, C. L. Mulder, N. N. Iossad, G. Rastelli, S. Ciuchi, A. F. Morpurgo, Tunable Fröhlich polarons in organic single-crystal transistors. *Nat. Mater.* **5**, 982–986 (2006).

67. W. Xie, K. A. McGarry, F. Liu, Y. Wu, P. P. Ruden, C. J. Douglas, C. D. Frisbie, High-mobility transistors based on single crystals of isotopically substituted rubrene-d_{28}. *J. Phys. Chem. C* **117**, 11522–11529 (2013).

68. J. Wang, R. M. Wolf, J. W. Caldwell, P. A. Kollman, D. A. Case, Development and testing of a general amber force field. *J. Comput. Chem.* **25**, 1157–1174 (2004).

69. I. Bulgarovskaya, V. Vozzhennikov, S. Aleksandrov, V. Belsky, *Latv. PSR Zinat. Akad. Vestis Khim. Ser.* 4, 53 (1983).

70. O. G. Ziogos, A. Kubas, Z. Futera, W. Xie, M. Elstner, J. Blumberger, HAB79: A new molecular dataset for benchmarking DFT and DFTB electronic couplings against high-level ab initio calculations. *J. Chem. Phys.* **155**, 234115 (2021).

71. T. Nematiaram, A. Troisi, Modeling charge transport in high-mobility molecular semiconductors: Balancing electronic structure and quantum dynamics methods with the help of experiments. *J. Chem. Phys.* **152**, 190902 (2020).

72. S. Ciuchi, S. Fratini, D. Mayou, Transient localization in crystalline organic semiconductors. *Phys. Rev. B* **83**, 081202 (2011).

73. T. Nematiaram, S. Ciuchi, X. Xie, S. Fratini, A. Troisi, Practical computation of the charge mobility in molecular semiconductors using transient localization theory. *J. Phys. Chem. C* **123**, 6989–6997 (2019).

74. T. C. P. Chui, D. R. Swanson, M. J. Adriaans, J. A. Nissen, J. A. Lipa, Temperature fluctuations in the canonical ensemble. *Phys. Rev. Lett.* **69**, 3005–3008 (1992).
